# A Scalable Perovskite Platform With Multi‐State Photoresponsivity for In‐Sensor Saliency Detection

**DOI:** 10.1002/adma.73243

**Published:** 2026-04-29

**Authors:** Xuechao Xing, Anubhab Tripathi, Si En Ng, Natalia Yantara, Yue Gong, Qinjie Wu, Yeow Boon Tay, Wen Siang Lew, Yang Chai, Arindam Basu, Nripan Mathews

**Affiliations:** ^1^ School of Materials Science and Engineering Nanyang Technological University Singapore Singapore; ^2^ Department of Electrical Engineering City University of Hong Kong Hong Kong China; ^3^ Energy Research Institute @ NTU (ERI@N) Nanyang Technological University Singapore Singapore; ^4^ Interdisciplinary Graduate School Nanyang Technological University Singapore Singapore; ^5^ School of Physical and Mathematical Sciences Nanyang Technological University Singapore Singapore; ^6^ Department of Applied Physics The Hong Kong Polytechnic University Hong Kong China

**Keywords:** face detection, halide perovskites, in‐sensor computing, ion migration, reconfigurable photoresponsivity

## Abstract

Artificial vision systems are increasingly central to edge intelligence, yet they often suffer from high data latency and energy consumption due to sensor‐processor separation. In‐sensor computing (ISC) provides a promising solution by integrating sensing and computation. However, current ISC devices remain constrained by scalability, uniformity, and processability. Here, we address these limitations via a reconfigurable perovskite‐photovoltaic platform that can be facilely processed from solutions. This architecture allows precise, reconfigurable photoresponsivity tuning with ultra‐low variability and supports fabrication on both rigid and flexible substrates. The device exhibits up to ±1120 mA W^−^^1^ photoresponsivity and 1000 programmable states, with excellent air stability (30 days) and thermal reliability (80°C). The scalability of these devices is demonstrated via a proof of concept 32 × 32 array. The excellent uniformity and programmability of the array are utilized in energy‐efficient face detection applications (achieving 95.2% sensitivity and 4.51 × speedup for subsequent computation) in addition to image feature extraction and MNIST digit recognition tasks (96.97% accuracy). Compared to previous ISC implementations, our system offers enhanced tunability, fabrication scalability, and functional stability. These results establish a practical perovskite‐based ISC platform, offering new avenues for intelligent computing systems in robotics, wearable electronics, and neuromorphic vision.

## Graphical abstract

A scalable in‐sensor computing platform (32 × 32 array) with ultra‐low variability is developed by incorporating ferroelectric copolymers into halide perovskite thin films. These devices achieve 1000 programmable photoresponsivity states and high thermal reliability. The system enables efficient parallel optical convolution for image processing, demonstrating high‐accuracy digit recognition and significant computational speedup for face detection in edge intelligence applications.

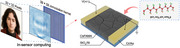

## 1 Introduction

The rapid evolution of autonomous vehicles, drones, and robotics has significantly increased the demand for highly efficient sensing and signal processing [1, 2, 3]. However, traditional computing architectures lead to data transmission bottlenecks and high energy consumption problems due to the physical separation of functional units. Therefore, architectures that can integrate different modules (sensing, memory, computing) need to be developed, enabling high‐performance, energy‐efficient machine vision for next‐generation autonomous systems [4, 5, 6, 7].

Although near‐sensor computing architectures have been explored to mitigate data transmission bottleneck (von Neumann Bottleneck), the separation of the sensing and the computing layers still limits processing efficiency [6, 8, 9]. Therefore, researchers have developed in‐sensor computing (ISC) architectures, which enhance sensing information processing efficiency by utilizing tunable photoresponsivities as weights for multiplication and accumulation operations in artificial neural networks [8, 9, 10, 11, 12, 13, 14, 15]. To enable this, a variety of optoelectronic devices exhibiting tunable photoresponsivity have leveraged mechanisms such as tunable density of vacancies [16, 17, 18] as well as dynamic charge trapping effects in device architectures employing multi‐gates [13, 14] bulk photovoltaic effect [11, 15] as well as vdW heterostructures [10]. However, such demonstrations of in‐sensor computing have been limited to single devices or small‐scale arrays (< 27 devices)‐ Table S1 [1, 10, 12, 13, 14, 15, 16]. In addition to suffering from device‐to‐device variability, these devices often exhibit limited photoresponsivity modulation due to the carrier transport dynamics in atomic‐scale thin layers, resulting in a few discrete states (7‐82 photoresponsivity states) [1, 7, 9, 12, 13, 14, 15, 16, 17, 18, 19]. Therefore, realizing a processable and scalable ISC platform with sufficient reconfigurable photoresponsivity remains an unsolved challenge.

Halide perovskites are a family of semiconductors that offer exceptional optoelectronic performance [20, 21, 22], tunable ion migration upon electric field [23, 24, 25, 26, 27], and facile solution processability [28, 29, 30], Benefitting from these advantages, halide perovskites can be considered as strong candidates for realizing tunable photoresponsivity in a scalable ISC platform. However, predictably regulating the ion migration has remained elusive [31]. Recent studies have incorporated ferroelectric polymers such as P(VDF‐TrFE) into perovskite photodetectors systems to create a remanent built‐in voltage that enhances photocarrier separation or to introduce pyroelectric‐photovoltaic coupling for improved responsivity and position‐sensitive detection [32, 33]. While these strategies effectively enhance the static photodetector metrics, the impact of ferroelectric polymers on programmable photoresponse modulation remains under‐explored, particularly regarding their transient photocurrent dynamics for computing applications. In addition, some studies focused on either suppressing ion migration to stabilize solar cells [34, 35, 36] or exploiting ion migration for analog behavior in memristive devices [37, 38], without addressing the critical needs for programmable and stable ISC devices [39, 40, 41].

Herein, we report a conceptually distinct strategy that transforms electric‐field‐driven ion redistribution in halide perovskites into a programmable computing primitive and stabilize the states by incorporating an engineered ferroelectric copolymer (EFC). This elegant and powerful solution‐processed approach enables fabrication of a 32 × 32 device array with ultra‐low device‐to‐device variability, demonstrating advantages in processability and scalability. The devices show precise modulated photoresponsivity across an ultra‐wide range of ±1120 mA W^−^^1^ with approximately 1000 programmable and distinguishable states‐ significantly surpassing the performance metrics of previously reported ISC devices [8, 9, 10, 11, 12, 13, 17]. This exceptional performance results in high accuracy in‐sensor classification of MNIST digits (>95% accuracy). Moreover, a cascade‐classifier‐based face‐detector combining the in‐sensor detector achieves 95.2% sensitivity on a popular face classification database (FDDB database), while only 22.2% patches are judged as “Face” and will be processed for downstream tasks, enabling over 4.51 × reduction in subsequent computation. This can be further enhanced by using multiple in‐sensor processors trained similarly to cascade classifiers. Finally, our devices demonstrate full compatibility with both rigid (SiO_2_/Si) and flexible (PET) substrates, which further broadens the application scenarios of this perovskite‐based ISC architecture. These findings indicate the considerable potential of our strategy for the realization of an intelligent and energy‐efficient platform for future sensory computing applications.

## 2 Results and Discussion

### 2.1 Reconfigurable Photovoltaic Behavior in Perovskite Devices

**FIGURE 1:**
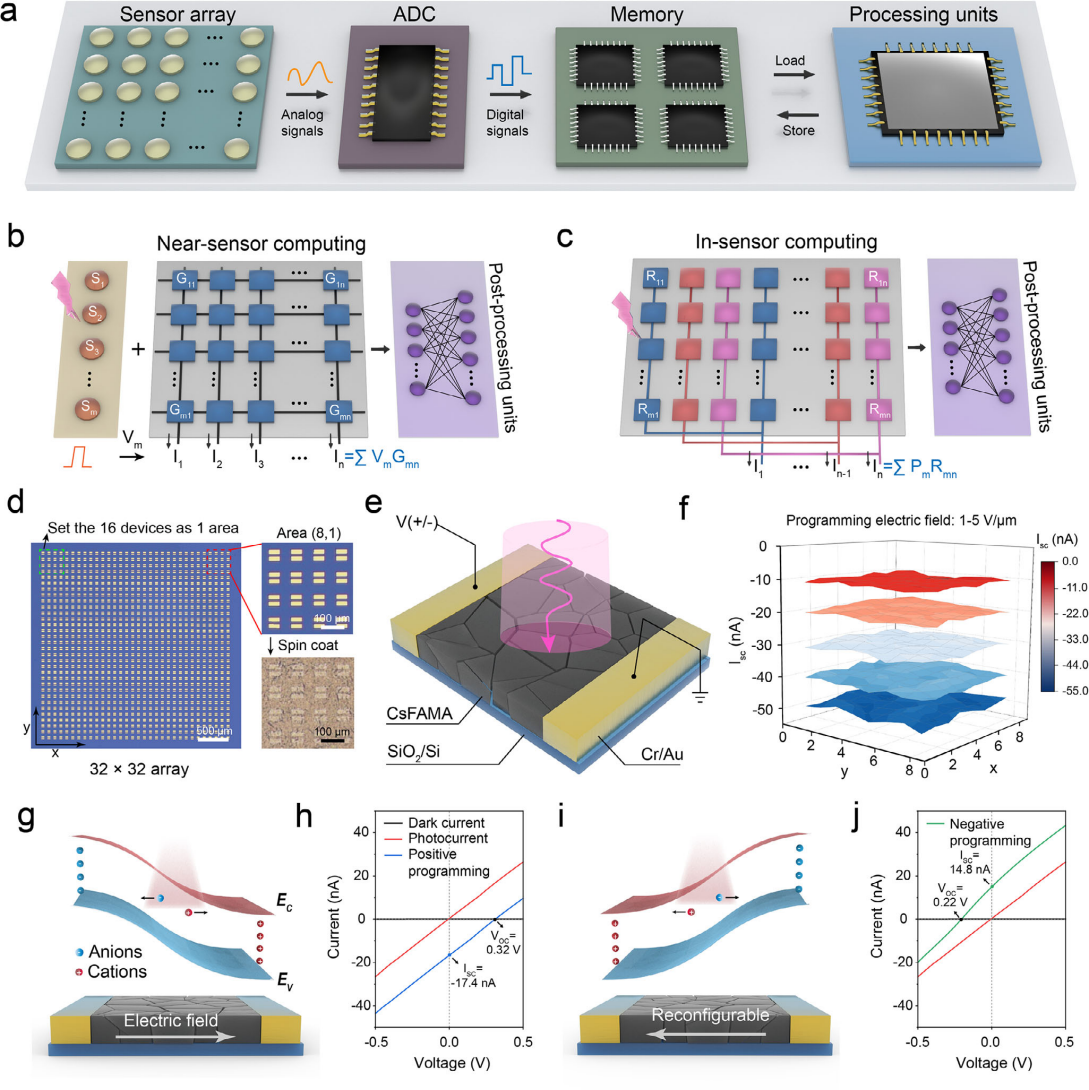
Architectures of sensory computing and demonstration of reconfigurable photovoltaic behavior in perovskite arrays. (a) Schematic of conventional sensory computing architecture. (b) Near‐sensor computing architecture with disconnected sensor layers and a front‐end processing unit. (c) In‐sensor computing architecture: The device has reconfigurable photoresponsivity and can be used for MAC operations in neural networks, receiving optical signals, and outputting electrical signals. (d) The optical image of the scalable array, which contains 32 × 32 individual devices, Top right image shows the zoom‐in area before spin coating, bottom right shows the zoom‐in area shows the area after spin coating. Each single lateral device with an electrode length of 50 um and a gap width of 10 µm. (e) Schematic diagram of the testing device, applying voltage or reading photocurrent through two electrodes. (f) Evaluation of device variability across the array. The device array was divided into 64 regions (each 4 × 4), and one random device per region was selected for testing. The I_sc_ across different regions exhibits a uniform and well‐distinguished distribution under various programming electric fields. (g) Schematic illustration of the ion migration‐induced photovoltaic effect. The redistribution of ions under an electric field establishes an internal potential gradient, enabling changes in its photovoltaic response. Photogenerated carriers separate in this structure and cause a photovoltaic effect (h). (i) The reverse voltage can reconfigure the ionic distribution, resulting in a reversible photovoltaic effect (j).

Traditional von Neumann architectures inherently suffer from data transfer bottlenecks due to the physical separation of computation and memory units (Figure 1a) [42]. Near‐sensor computing (NSC) integrates computing and memory functionalities to mitigate this issue, reducing the need for frequent data transfers. However, NSC still operates with discrete optical sensors and memristive computing units, requiring data movement between sensing and computing layers, introducing latency and energy inefficiencies (Figure 1b) [1, 6]. In contrast, ISC directly integrates optical signal detection, storage, and computation within a single functional unit (Figure 1c), minimizing data transfer latency and enhancing overall computational performance. In this system, photoresponsivity (R) serves as a computational weight, modulating optical input into electrical output with precise tunability. Perovskite materials, recognized for their superior optoelectronic properties, have been explored for their reconfigurable photovoltaic behavior, making them promising candidates for realizing ISC architectures [6, 43].

Motivated by these promising advantages, we fabricated a perovskite‐based reconfigurable photovoltaic array of 32 × 32 devices on SiO_2_/Si substrate. Each device adopts a lateral two‐terminal configuration (top right image) as illustrated in Figure 1d. The perovskite thin film is deposited via a spin‐coating process onto Au electrodes (bottom right image). Figure 1e shows the device architecture, featuring a triple cation (CsFAMA) perovskite thin film on a SiO_2_/Si substrate with Cr/Au metal contacts. To evaluate the uniformity of the 1024 devices, we employed a representative sampling strategy, selecting devices from 64 distinct regions for characterization. The results, presented in Figure S1 and Figure 1f, show that the short‐circuit current (I_sc_) across different regions exhibits a uniform and well‐distinguished distribution under various programming electric fields. This high integration density and low device‐to‐device variability in a perovskite‐based array significantly surpasses what has been reported in ISC applications, highlighting the superior scalability and processability of our approach (Table S1). The reconfigurable photovoltaic behavior in such a symmetric device configuration is consistent with electric field‐induced ion redistribution, enabling dynamic tunability of the local potential. Perovskite materials, characterized by their soft lattice framework, contain mobile halide vacancies (e.g., I^−^ vacancies) and organic/inorganic cations (e.g., MA^+^, FA^+^). In general, grain boundaries and defects are often considered the primary pathways for ion migration in perovskite film (Figure S2a), as reported in prior studies [44, 45]. Moreover, by applying an external electric field, ion redistribution can be induced, leading to changes in the device's photovoltaic parameters in a reversible manner, such as I_sc_ and open‐circuit voltage (V_oc_) [27]. This inherent ion migration modulation phenomenon provides a convenient means to adjust the device's photovoltaic response and thus achieve reconfigurable photoresponsivity.

To investigate how electrical conditioning influences the photovoltaic behavior, we modulated the perovskite thin film by applying an external electric field. The resultant ion migration is expected to modulate the internal potential profile, illustrated conceptually in Figure 1g. Under this condition, we illuminated the device while performing small voltage sweeps (−0.5 to 0.5 V) to measure the photocurrent (Figure 1h). Subsequent to electric field programming, the device exhibited a pronounced V_oc_ = 0.32 V, demonstrating the modulation of the photovoltaic effect. This effect is reversible, as shown in Figure 1i, where a counter electric field results in an inverse photovoltaic response, confirming the reconfigurability of the device (Figure 1j). However, this programmable photovoltaic state is not permanently retained. Due to spontaneous ion relaxation upon field removal, the photovoltaic characteristics gradually decay over time, consistent with ionic dynamics reported in perovskite materials. Figure S2b presents the relaxation of I_sc_, revealing that both positive and negative I_sc_ exhibit approximately 70% decay within a minute, highlighting the need for kinetic stabilization.

### 2.2 Enhanced Linear Programmability and Environmental Stability via EFC Incorporation

**FIGURE 2:**
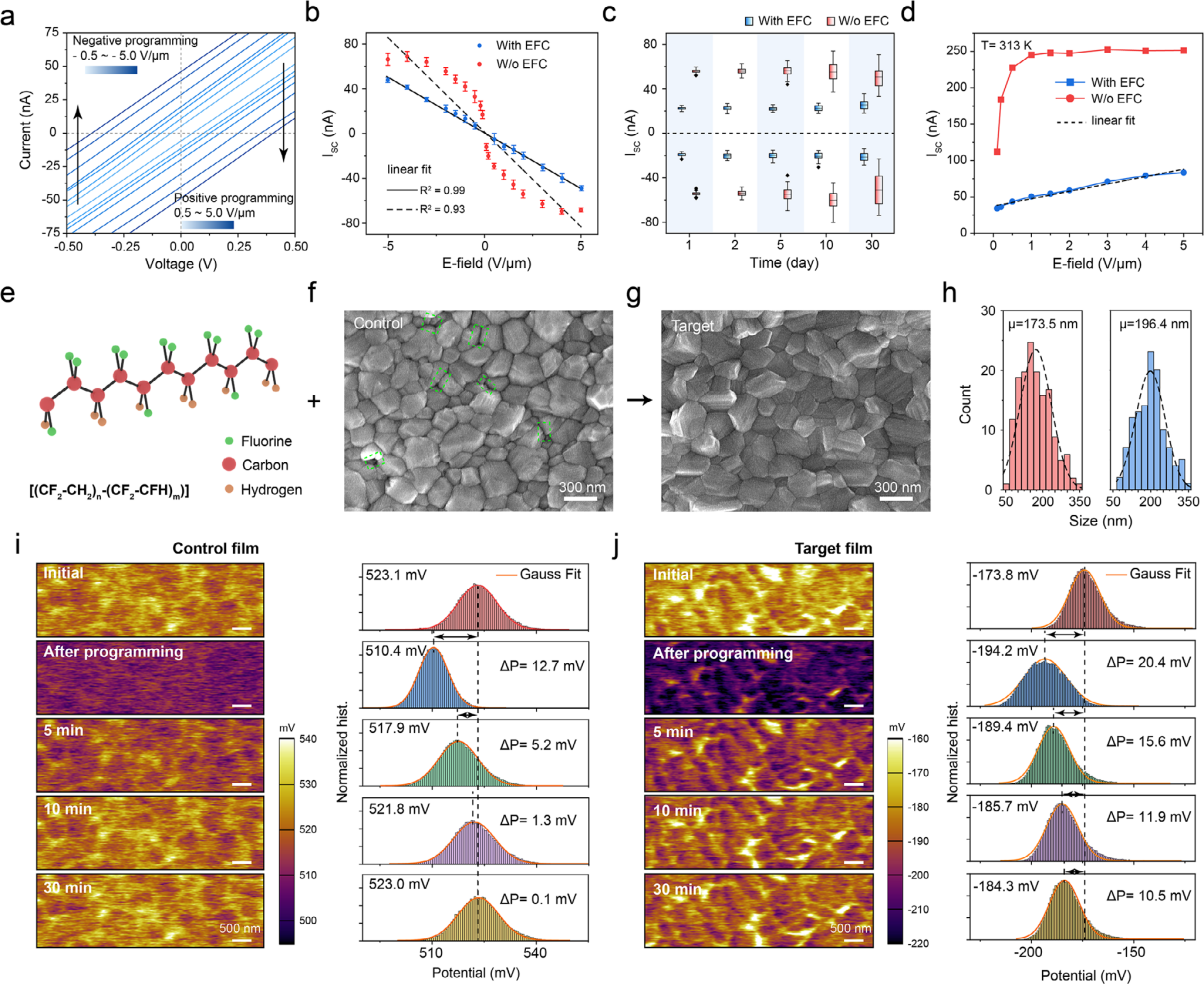
Electrical tuning and stability characteristics of perovskite devices with and without EFC incorporation. (a) Reconfigurable photovoltaic effect of the target device after each positive and negative programming, sweeping rate of 0.05 V/step, illumination intensity:50 mW cm^−2^, light wavelength: 623 nm. (b) Comparison of I_sc_ between the control and target device under different programming conditions. The distribution is from the test of 5 devices. (c) Stability test of control and target device in an atmospheric environment, storage time is 1, 2, 5, 10, 30 days. I_sc_ distribution statistics of 25 devices. Programming electric field: 2 V/µm. (d) I_sc_ of the control device and target device at 313 K under different programming conditions. (e) Structure diagram of ferroelectric polymer. (f) Top‐view SEM images of the control and (g) target samples, corresponding to the grain size distributions of the (h). (i) KPFM images (left) and surface potential distribution statistics (right) of the control sample at different times after programming. (j) KPFM images (left) and surface potential distribution statistics (right) of the target sample at different times after programming.

We investigated reconfigurable photovoltaic performance under different electrical programming fields. Figure S3 shows the *I‐V* curves of the control samples under different programming electric fields. It is worth noting that the control sample generated observable V_oc_ and I_sc_ under a relatively low electric field of 0.1 V/µm, indicating a low threshold for ion motion in the perovskite film. We gathered statistics on the I_sc_ of the five devices under different programming electric field conditions (ranging from −5 to 5 V/µm). As shown in Figure 2b, the linear relationship between I_sc_ and the electric field of the control sample is not ideal (R^2^ = 0.93), indicating that the electrical tuning range of the pristine perovskite film is relatively limited. To get a more reliable reconfigurable photovoltaic device, we incorporated EFC (0.5 mg/mL) into the perovskite precursor solution to optimize the retention characteristic, as illustrated in Figure 2e. EFC is a fluoropolymer that has been demonstrated to influence interfacial electronic properties in perovskite solar cells, facilitating carrier separation and improving photovoltaic efficiency [46, 47]. Prior studies suggest that fluoropolymer additives may interact with perovskite surfaces or defect sites, contributing to modified interfacial environments and improved stability [48, 49]. In Figure S4a,b, we further evaluated the effect of EFC incorporation by performing UV–vis absorption and steady‐state photoluminescence (PL) for films with different EFC concentrations (0–3 mg mL^−1^). The absorption edges remain nearly unchanged, indicating that the band structure of the perovskite is largely preserved upon polymer incorporation. Meanwhile, the 0.5 mg mL^−1^ film exhibits the strongest PL emission, suggesting improved film quality at moderate polymer loading. Based on this, we checked the reconfigurable photovoltaic performance of the target sample to assess the effect of EFC incorporation. Figure 2a shows the results of the target samples with different programming electric fields. Compared to the control sample, the EFC‐incorporated film required a higher field strength (0.5 V/µm) to reach comparable photovoltaic outputs, which results from the insulating nature of the EFC polymer and the modified transport environment. However, this allows for linear programmability (R^2^ = 0.99) as indicated in Figure 2b. It should be noted that, unlike conventional perovskite solar cells where the ferroelectric incorporated‐perovskite is integrated with electron and hole transport layers to maximize power conversion efficiency, the present device adopts a lateral two‐terminal architecture with an electric‐field‐programmed mechanism. In this configuration, the role of the EFC additive is not to maximize V_oc_ or I_sc_ output but to regulate ionic migration dynamics and stabilize the programmed photovoltaic states.

Additionally, the incorporation of EFC long chains has been reported to mechanically reinforce the perovskite film, thereby improving phase stability against thermal fluctuations and mechanical stress [50]. As shown in Figure 2c, the target device exhibited a stable I_sc_ distribution even after 30 days, while the I_sc_ distribution of the control device gradually broadened, indicative of degradation under environmental exposure. Furthermore, temperature increases substantially influence the electrical response of perovskite films, adversely affecting I_sc_. At an elevated temperature of 40°C (313 K), both samples exhibited increased currents, as shown in Figure 2d. However, the I_sc_ of the control device rapidly reached saturation as the programming electric field increased. In contrast, the target device can still maintain the linear relationship between I_sc_ and the programming electric field, highlighting improved operational stability under elevated temperature conditions. We further evaluated the thermal cycling stability of the devices under varying temperature conditions, as shown in Figure S5. The devices were exposed to temperatures ranging from 50°C to 80°C in ambient air for 15 min, after which their I_sc_ was measured at room temperature. The results reveal the I_sc_ of the control device exhibits an increasing trend with rising temperature, while the target device remains consistently stable across the temperature range, consistent with its more stable electrical characteristics. These findings indicate the great potential of the device, suitable for real‐world, complex operational environments. To evaluate the possibility that EFC alone contributes to the photovoltaic effect, we fabricated a reference device with EFC as the active layer. As shown in Figure S6, no measurable photovoltaic response or significant photocurrent (∼pA) was observed after electric field programming, which supports that the observed reconfigurable photoresponse originates primarily from the perovskite film.

We characterized the morphology of the control and the target sample (mixed with EFC). As shown by the scanning electron microscopy (SEM) image (Figure 2f), the control perovskite film exhibits obvious gaps at the grain boundaries, which are widely recognized as favorable channels for ion transport in polycrystalline films [50, 51]. Statistical analysis of grain sizes in this area (Figure S7) reveals an average grain size of 173.5 nm (Figure 2h). In contrast, incorporation of EFC film exhibits a more compact morphology compared to the control sample (Figure 2g), and the corresponding grain size increases to an average value of approximately 196.4 nm. SEM images of films prepared with higher EFC concentrations (1–3 mg mL^−1^) are provided in Figure S4c for comparison. The improved surface quality of the target sample is further supported by atomic force microscopy (AFM) results (Figure S8), which show a reduced surface roughness of 33.0 nm compared to 39.12 nm of the control film.

Building upon the morphological improvements observed in the target films, we next monitored changes in the surface electrostatic potential. Ion migration involving mobile halide ions (I^−^, Br^−^) and organic cations (MA^+^, FA^+^) in perovskite films alters the local surface potential, making it possible to indirectly probe ionic dynamics using Kelvin Probe Force Microscopy (KPFM) [24, 50]. Here, we employed KPFM by monitoring changes in contact potential difference (CPD) before and after electrical field programming. The programming electric field is applied laterally along the electrode gap direction, consistent with the planar two‐terminal device architecture. Under such lateral bias, mobile ions redistribute horizontally, giving rise to an internal potential gradient across the channel, which constitutes the physical origin of the switchable photovoltaic response (Figure S9). However, since the present study focuses primarily on the local relaxation behavior of the programmed state, the KPFM scan area was intentionally reduced to 5 µm × 5 µm to minimize the influence of large‐scale potential gradients. For clarity and layout considerations, only a representative subregion (∼5 µm × 1.8 µm) is displayed in Figure 2i–j, while the complete 5 µm × 5 µm maps are provided in the Figure S10. As illustrated in Figure 2i, the control sample showed a potential change(ΔP) of 12.7 mV after programming, showing a noticeable change in surface potential after programming. However, the potential rapidly relaxed to nearly its original value (ΔP ∼ 1.3 mV at 10 min), showing limited retention. In contrast, the target sample (Figure 2j) demonstrated a larger initial ΔP of 20.4 mV, which decayed more gradually and maintained a residual ΔP of 10.5 mV after 30 min. This substantial long‐term potential offset indicates that the EFC‐perovskite hybrid system possesses significantly slower ionic relaxation kinetics, thereby stabilizing the field‐induced charge distribution. This interpretation is further supported by device‐level retention measurements in Figure S11, the retention behaviour of the dark current and the I_sc_ were checked both on the control and the target device. The programmed photoresponsivity state in the target sample can maintain, whereas the control sample relaxes much more rapidly toward its initial state, consistent with the KPFM‐observed potential evolution.

### 2.3 Electromechanical Response of EFC‐Incorporated Perovskite Films and Conceptual Schematic

**FIGURE 3:**
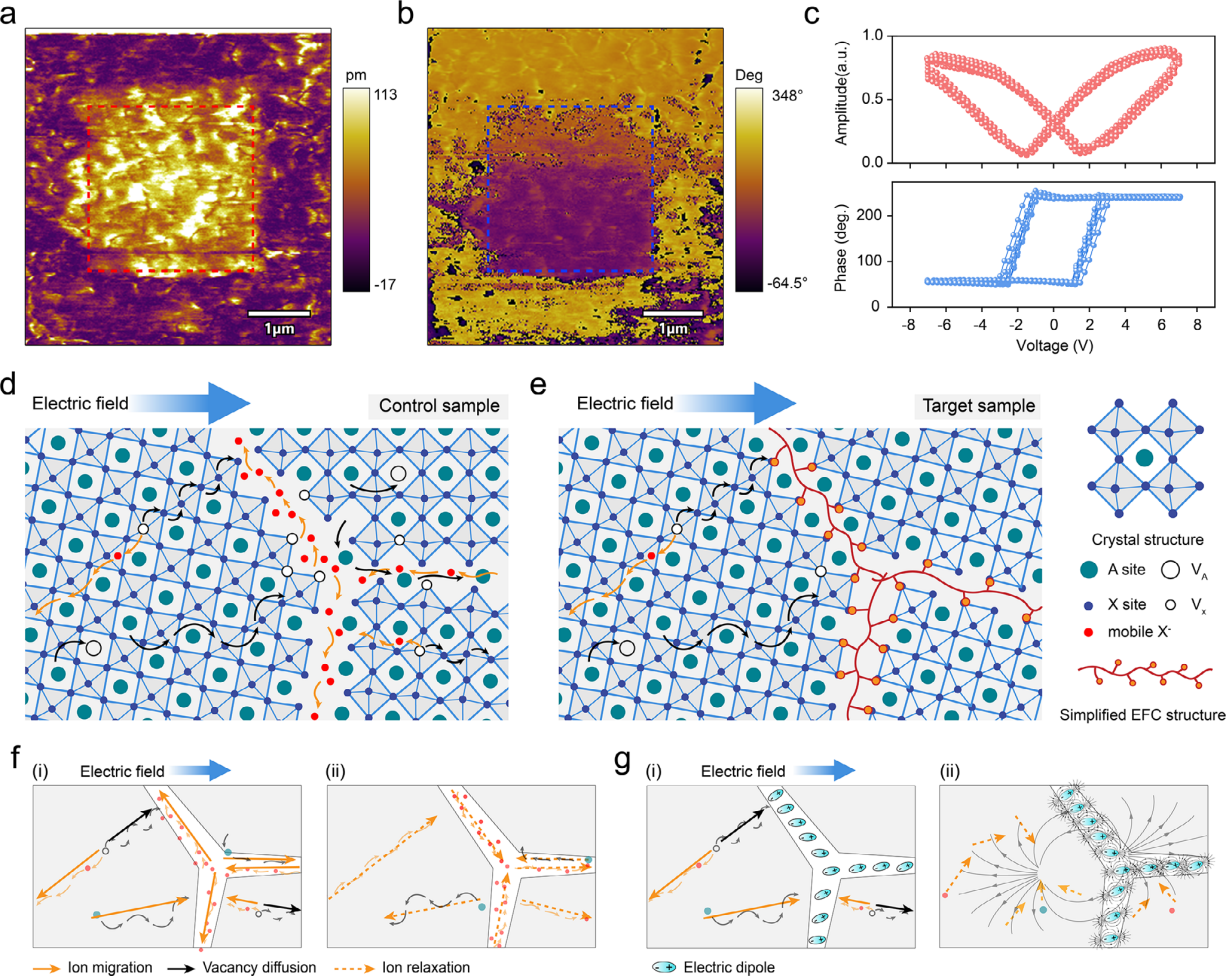
Electromechanical response and conceptual schematic of electrical conditioning in pristine and EFC‐incorporated perovskite films. (a) In‐plane polarization amplitude and (b) phase images of EFC‐incorporated perovskite film after electrical poling. (c) Butterfly‐shaped amplitude‐voltage hysteresis (red) and Phase‐voltage hysteresis (blue) loops measured by PFM, showing consistent switching behavior over multiple consecutive cycles. (d) Conceptual schematic illustrating possible field‐induced redistribution processes in the control sample. (e) Illustration of the ionic migration pathways in the perovskite film with EFC under the influence of an electric field. The presence of long chains of EFC in the grain boundaries reduces the available paths for ion migration. (f) Simplified depiction of electrically induced redistribution and subsequent relaxation under no external bias for the control sample. (i). Under the influence of an electric field, ions migrate to align with the electric field. (ii). Upon removal of the electric field, ions tend to return to their original state. (g) Schematic diagram of ion migration pathway in the target sample. (i). Under the influence of an electric field, the grain boundaries traditionally serve as pathways for ion migration, are occupied by long chains of EFC. The dipoles tend to align with the direction of the field. (ii). Upon removal of the electric field, ions are unable to return to their original positions effectively due to the influence of the residual ferroelectric field.

Beyond morphological effects, the introduction of EFC imparts ferroelectric functionality to the hybrid film, providing an additional mechanism to influence their electrical response. To verify the presence of polarization and its potential role, we further examined how electrical conditioning influences the electromechanical response of the films by performing piezoresponse force microscopy (PFM) on the control and target samples. For the control sample (Figure S12), no discernible contrast was observed between electrically poled and unpoled regions under a DC bias of ±8 V. In contrast, the EFC‐incorporated film exhibited clear amplitudes and phase contrasts within the poled region (Figure 3a,b). Also, the amplitude‐voltage and phase‐voltage curves of the target sample exhibit a typical butterfly curve shape and a clear 180° phase switching hysteresis loop over repeat bias sweeps, as shown in Figure 3c. These characteristic signatures confirm the existence of reversible polar ordering within the hybrid film at room temperature of the target sample, distinct from conventional β‐phase ferroelectricity, but rather as field‐activated and partially stabilized dipolar alignment within the polymer‐perovskite hybrid matrix.

To examine whether polymer incorporation introduces structural modifications, we analyzed the Grazing‐Incidence Wide‐Angle X‐ray Scattering (GIWAXS) patterns. Notably, the target films exhibit diffraction features nearly identical to the control (Figure S13), confirming that the trace EFC incorporation preserves the high‐quality bulk perovskite lattice without inducing distortion. This structural preservation strongly implies that the EFC molecules do not intercalate into the bulk grains but rather selectively segregate at the grain boundaries, which are known to serve as dominant ion migration pathways. Based on these characteristics, we propose a conceptual model to interpret the possible behaviors observed in pristine and EFC‐incorporated perovskite films. In a perovskite film (Figure 3d), ionic migration occurs through multiple pathways under external electric fields, such as point defects and grain boundaries. Within the lateral perovskite structure, electric‐field‐driven ion redistribution establishes a nonequilibrium internal potential gradient. This gradient is reconfigurable and programmable, depending on the polarity and magnitude of the applied field [24]. Importantly, this internal potential gradient acts to spatially separate photogenerated carriers along the lateral direction, thereby giving rise to the corresponding photovoltaic response. After removing the field, the redistributed ions spontaneously relax back to their equilibrium state due to concentration gradients (Figure 3f(i,ii)), leading to the rapid decay of the programmed photovoltaic state. For the target sample (Figure 3e), the more compact morphology (Figure 2g) and significantly reduced dark current (Figure S14) indicate a modified electrical environment relative to the control sample. Thus, under an applied electric field, only limited potential channels are available for ion migration (Figure 3g(i)), thereby restricting rapid ionic transport. So, the incorporation of the EFC plays a stabilizing role that regulates ionic transport and suppresses fast diffusion pathways rather than merely enhancing the separation of photogenerated carriers. After removal of the external bias, this internal field opposes the diffusive relaxation of ions, thereby stabilizing the non‐equilibrium state and enhancing the retention of the photovoltaic effect (Figure 3g(ii)). Collectively, unlike previously reported ferroelectric‐assisted perovskite devices that rely primarily on static polarization fields or transient pyroelectric coupling to enhance carrier separation [32, 33], the present system deliberately harnesses ion migration and stabilizes its nonequilibrium distribution, with a reversible programmable ability. This mechanism transforms ion migration from a detrimental instability factor in most of the perovskite devices into a controllable and functional degree of freedom for ISC systems.

### 2.4 Reconfigurable Photoresponsivity Characteristics and Optoelectronic Performance

**FIGURE 4:**
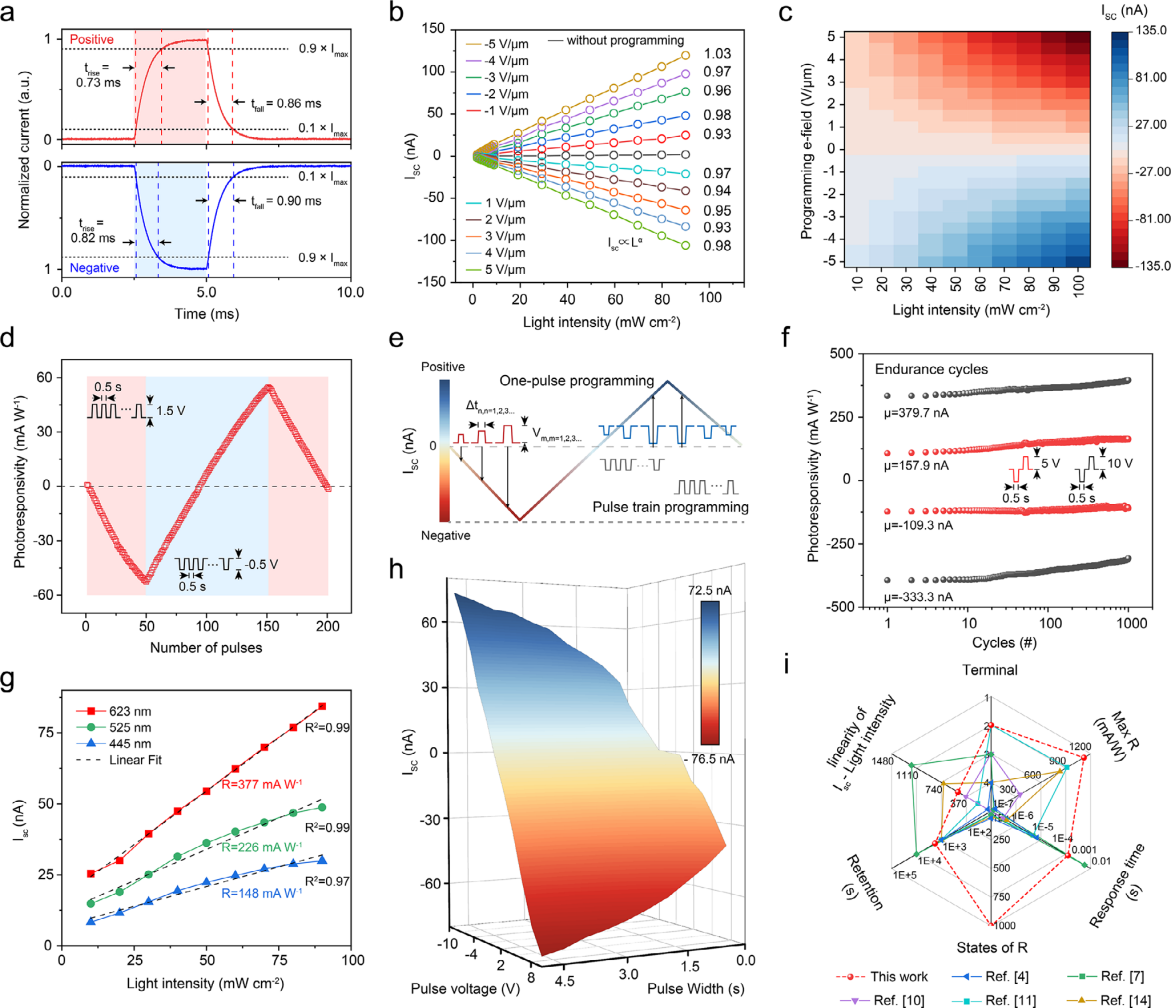
Reconfigurable photoresponsivity characteristics and optoelectronic performance of target devices. Reconfigurable photoresponsivity of the target device. (a) Transient photocurrent rise and fall of the device in positive and negative photo response states. The input irradiance is 50 mW cm^−2^ (623 nm). The light pulse is triggered on (rise)/off (fall) at time t = 0.0025 s/0.005 s. (b) The linear dependence of I_sc_ vs. light intensity under positive and negative programming electric field. (c) Dependence of I_sc_ on different programming electric fields and illumination intensity. (d) Linear and reconfigurable photoresponsivity updates with 200 pulses. For Stages I and III, 50 pulses with 1.5 V amplitude and 0.5 s width were used. For stage II, 100 pulses with −0.5 V amplitude and 0.5 s width were used to engineer the same slope for positive and negative weight updates. All the levels were read under 623 nm light when the pulse is low level (0 V). (e) Schematic of one‐pulse programming. The photoresponsivity level programmed can also be accessed using single‐pulse programming by changing the amplitude and the pulse width. (f) Endurance cycles of photoresponsivity under the positive and negative pulses. The voltage amplitudes of the pulses are ±5 and ±10 V, and the pulse width is 0.5 µs. (g) Wavelength‐dependent of the I_sc_. The goodness of fit (R^2^) is close to 1, demonstrating the linear correlation. (h) The I_sc_ value responds to a single pulse with various widths and amplitudes. (i) Radar graph comparing the main parameters of the devices between this work and other emerging reconfigurable photoresponsivity. The number of “terminals” reflects the complexity of the device structure. Linearity of I_sc_ ‐light intensity indicates the light intensity range for the device to maintain a linear response.

To construct an ISC architecture based on the EFC incorporated films, it is essential to first evaluate its fundamental optoelectronic characteristics. The device inherently operates without additional energy input during sensing, as the photovoltaic effect drives its fundamental operation. As shown in Figure 4a, the transient photoresponse was measured under zero‐bias (short‐circuit) conditions using a modulated 623 nm light source. The device exhibited a rise time of approximately 0.8 ms and a decay time of 0.9 ms, defined as the time required for the photocurrent to change from 10% to 90% (and vice versa) of its steady‐state value. To quantitatively evaluate this behavior, the intensity‐dependent I_sc_ was analyzed using a power‐law relationship, *I_sc_* ∝ *L* ^α^, where *L* denotes the incident light intensity and α is the light‐response exponent [51]. As shown in Figure 4b, the result revealed a dependence between I_sc_ and incident light intensity across 3–90 mW cm^−2^ light intensity under various programming electric fields. The fitted α values range from 0.93 to 1.03, remaining close to unity for both positive and negative programmed states, which confirms that the photoresponse operates within a sublinear regime. Beyond the linearity of light response, the operational stability of each state is also critical, Figure S15a shows each corresponding photoresponsivity states remain clearly distinguishable over 1800 s under the 50 mW cm^−2^ light condition. Furthermore, cyclic measurements (Figure S15b–e) of light pulses exhibit negligible drift over 500 cycles, confirming that the programmed states are stable under continuous illumination. The device also maintains reliable responses even at light intensities up to 500 mW cm^−2^ (Figure S16a, b), further demonstrating its robustness and practical applicability under a wide range of operating conditions. Figure S16c illustrates intensity‐dependent I_sc_ under more programming conditions(0.92 < α < 1.04), corresponding to distinct photoresponsivity states(Figure S16d), which is already sufficient to meet the requirements of common convolution operations, such as the Sobel kernel utilizes only a limited set of discrete weights [11]. Further analysis of I_sc_ (Figure 4c) revealed symmetrical distributions under both positive and negative photoresponsivity conditions. Notably, these symmetrical photovoltaic responses allow enhanced precision in device control and reconfigurability. In contrast, as shown in Figure S17a, although the I_sc_ of the control sample is bidirectionally tunable, its dependence on light intensity exhibits lower linearity(0.80 < α < 0.88). This reduced linearity likely arises from the less stable electrically conditioned states in the control sample, which limits the precision of photoresponsivity tuning.

Since photoresponsivity directly functions as a tunable convolutional weight, achieving continuous and linear update characteristics in practical use is essential. To demonstrate the linearity of weight updates, we applied sequential voltage pulses. As illustrated in Figure 4d, using a pulse width of 0.5 s (amplitude 1.5 V) results in a linearly and continuously modulated negative photoresponsivity. Then, 100 square wave pulses of −0.5 V with a duration of 0.5 s can be applied to switch the photoresponsivity value linearly from negative to positive. Under the same test conditions, the control device showing less stable current responses, consistent with reduced controllability. (Figure S17b). It is notable that the amplitude and duration of pulses affect both the linearity and symmetry of the photoresponsivity updates. As shown in Figure S18a,b, when the write pulse width is decreased to 50 ms, the target device maintains a linear increase in photoresponsivity under pulse stimulation, achieving approximately 1000 accessible photoresponsivity states. To the best of our knowledge, this represents one of the highest resolutions of programmable photoresponsivity states reported to date (Table S2). Furthermore, benefiting from the intrinsically strong photoresponse performance of perovskite materials, adjusting the write pulse amplitude to +5 and −2.5 V enables the tuning of photoresponsivity values up to ±1120 mA W^−^^1^, the detail in Figure S18c,d. Beyond continuous pulse schemes, we investigated single‐pulse programming strategies (Figure 4e), systematically varying pulse amplitudes from −10 to 10 V (2 V interval) and durations from 0.5 to 5 s (0.5 s interval). Under the light intensity is 50 mW cm^−2^, I_sc_ can vary from −76.5 to 72.5 nA (Figure 4h), indicating that single pulse programming can achieve a range of ±700 mA W^−1^. This capability allows for coarse‐grained weight initialization followed by fine‐tuning, significantly accelerating the overall training latency. This result highlights the excellent reproducibility and precision of single‐pulse‐induced photoresponsivity states, with clear and discrete state distributions ranging from negative to positive regions.

For edge computing applications where convolutional kernels require periodic updating/reconfiguration, the stability of modulation is also crucial. As illustrated in Figure 4f, the device exhibited robust retention across 1000 consecutive programming cycles with no significant degradation or overlap between states, demonstrating stable operation across repeated programming cycles. To assess spectral adaptability, we evaluated the photoresponse at different wavelengths under 10–90 mW cm^−2^ light illumination (Figure S19; Figure 4g). I_sc_ remained highly linear across multiple wavelengths, supporting its potential for multiband image processing and convolution computing applications. Finally, a benchmarking comparison between our device and recent state‐of‐the‐art programmable photovoltaic devices (Figure 4i) highlights the strong performance of our system in terms of fabrication simplicity, photoresponsivity range, and the number of states. In the context of ISC systems, the device structure plays a critical role in determining both the fabrication complexity and power consumption; the maximum photoresponsivity value and the number of distinguishable states are crucial for weight encoding precision. Additionally, the ability to maintain a linear correlation between I_sc_ and light intensity is important for the applicability of the device in low‐light sensing and dynamic range extension. (A more detailed summary is provided in Table S2) Taken together, these performance metrics highlight the advantages of our platform in achieving high‐linearity, high‐resolution, and multi‐state photoresponsivity control, which is essential for next‐generation in‐sensor computing and neuromorphic applications.

### 2.5 Parallel in‐Sensor Optical Convolution and High‐Accuracy Neuromorphic Recognition

**FIGURE 5:**
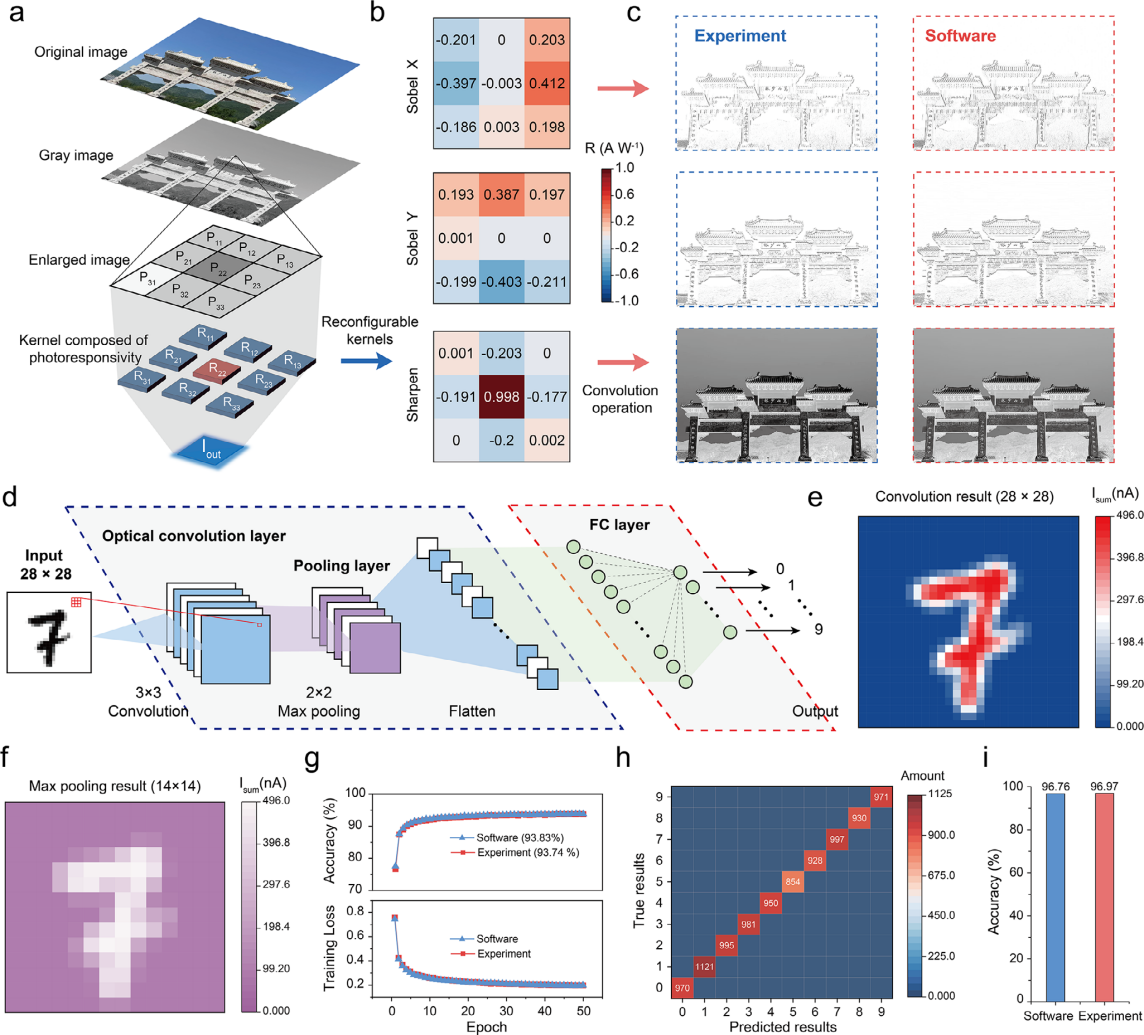
Experimental demonstration of optical convolution operations and MNIST digit classification using ISC arrays. (a) A flow chart diagram of a convolution operation on an optical signal input, the kernel is composed of reconfigurable photoresponsivity array, and the output is an electrical signal. (Original photograph taken by the author) (b) Three reconfigurable 3 × 3 kernels are defined by photoresponsivity, which can work simultaneously. The functions of Sobel X, Sobel Y, and Sharpen are to detect edges in the x direction, detect edges in the y direction, and sharpen the image, respectively. The unit of photoresponsivity is mA W^−1^. (c) The results of the convolution operation using experimental kernels and software‐generated kernels. (d) Schematic diagram of a convolutional neural network model for classifying handwritten digits, with the optical convolution layer performing feature extraction and the fully connected layer performing classification. (e) The edge features extracted from the number “7” with 623 nm light as input when the convolution kernel is a Gaussian kernel (R = [0.09, 0.22, 0.12] [0.19, 0.37, 0.21] [0.1, 0.2, 0.11]). The unit of R is mA W^−1^. Define light input 500 mW cm^−2^ as the corresponding grayscale maximum value. (f) Use max pooling (2 × 2) to enhance feature extraction. (g) Training accuracy and training loss of MNIST recognition. The convolution kernel in the experiment is trained offline, while the convolution kernel in the software can be updated. (h) The confusion matrix shows the accurate classification and misclassification using the experimental training results. (i) Comparison of the accuracy of MNIST classification using experimental and software training results.

To demonstrate the in‐sensor computing potential of our perovskite‐based reconfigurable photovoltaic device array, we directly implemented optical convolution operations for image processing tasks, including image sharpening, edge detection, feature extraction, and neural network construction. First, we demonstrate the device's ability to perform convolution processing on optical signals. Figure 5a describes the principle of optical convolution operation, in which the convolution kernels are implemented through programmed photoresponsivity values. To examine performance and substrate compatibility, we designed 27 device‐array on both hard (SiO_2_/Si) and flexible substrates (poly(ethylene terephthalate), PET) substrate, as shown in Figure S20a. The array is designed as a parallel processing module, containing multiple functional blocks that can be configured into three independent 3 × 3 convolution kernels simultaneously (Figure S20b). This design allows for the parallel extraction of distinct image features within a single sensing frame. Characterization results (Figure S20d–f) reveal substrate‐independent performance uniformity, confirming that the EFC‐modulated perovskite strategy is compatible with both rigid and flexible substrates. Moreover, bending tests on the flexible device at radii of 10 and 5 mm for up to 200 cycles show nearly unchanged programmed I_sc_ distributions (Figure S21), indicating good mechanical stability under repeated deformation. Before programming photoresponsivity into the devices, we checked their initial states and verified that the V_oc_ of all devices was close to zero, providing a stable and well‐defined baseline (Figure S22). Then, as shown in Figure 5b, we defined classic image processing kernels, including Sobel X (horizontal edge detection), Sobel Y (vertical edge detection), and sharpening kernels. Each kernel was implemented by assigning a specific photoresponsivity value (unit: mA W^−^^1^). Figure 5c shows that feature processing after the convolution operation is a close match with software‐simulated results, proving the efficacy of our proposed approach. Furthermore, the high tunability of photoresponsivity enables flexible customization of multiple convolution kernels, enabling versatile image processing tasks such as edge detection and denoising (Figure S23).

To benchmark the system‐level performance, we constructed a hybrid optoelectronic convolutional neural network (CNN) for MNIST digit classification (Figure 5d). In this architecture, the input image is first processed through an optical convolution layer (3 × 3 convolution kernel). Taking a Gaussian kernel for image smoothing and noise removal, Figure 5e shows the feature extraction result obtained for digit “7” image in the MNIST database. The conv2D layer was implemented with the same (zero) padding so that the output spatial dimensions match the input (28 × 28 pixels) and is then further processed through a pooling layer (2 × 2 max pooling), the pooled results (14 × 14 pixels) as shown in Figure 5f. Finally, to show the information content of the filtered image, the classification is performed by a fully connected (FC) layer, producing an output of ten categories (0–9). We performed 50 epochs of offline training on 60000 randomly selected MNIST images, maintaining fixed convolution kernel parameters and updating solely the weights in the FC layer, with results for training accuracy and loss illustrated clearly in Figure 5g. It should be noted that the training accuracy (93.74%) is recorded during optimization with dropout regularization enabled (Dropout = 0.5) in the fully connected layer, closely approaching the 93.83% accuracy attained in fully software‐based CNN training where an ideal Gaussian kernel was used. Whereas the final evaluation in Figure 5i is performed in inference mode with dropout disabled (Dropout = OFF). Therefore, the evaluation accuracy can be higher than the training accuracy. To further demonstrate the denoising effects of our kernel, we show the impact of introducing noise on images and classification accuracy in Figure S24. As the levels of noise introduced increase, the recognition accuracy of the systems using the ideal and experimental Gaussian kernels shows the same downward trend. When the introduced noise standard deviation is 0.2, the accuracy drops to about 47.05%, while the neural network without a Gaussian convolution kernel only maintains a recognition accuracy of 25.97%. The confusion matrix in Figure 5h further confirms robust classification performance across the entire 10 000‐sample MNIST test set, with an overall recognition accuracy exceeding 95%. Finally, Figure 5i compares the experimental MNIST classification accuracy (96.97%) with software simulations (96.76%, the corresponding confusion matrix shown in Figure S25), validating the reliability and effectiveness of the proposed system. Note that our approach can be used to program the first convolutional layer of any deep CNNs, which could achieve higher accuracy.

### 2.6 In‐sensor Saliency Filtering for Energy‐Efficient Face Detection

**FIGURE 6:**
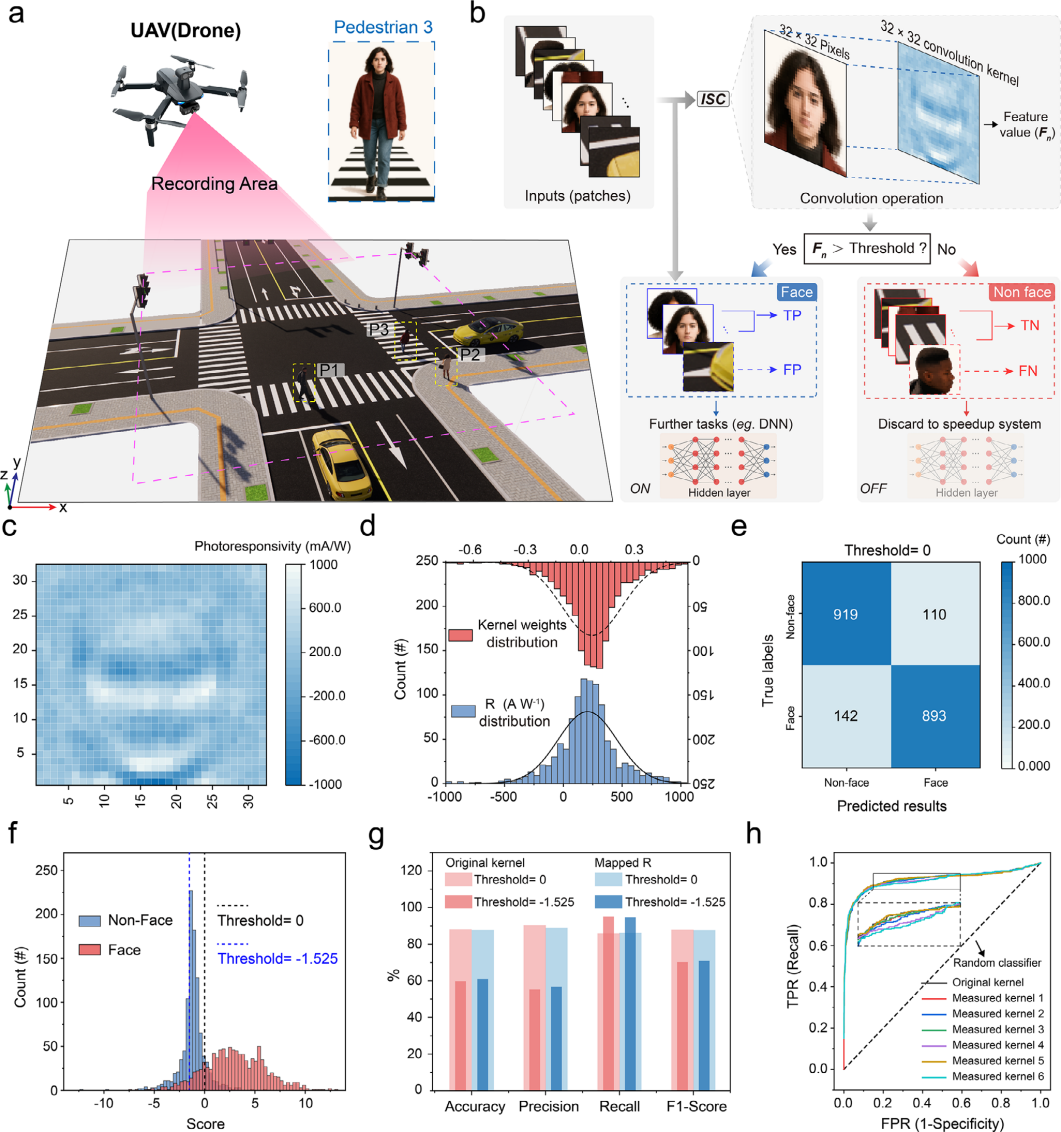
Single‐layer neuromorphic saliency filtering for face detection. (a) Schematic illustration of an application scenario where a UAV (unmanned aerial vehicle) equipped with a visual sensing system performs face detection at a road intersection. (b) Schematic diagram of the face/non‐face classification using a 32 × 32 device array as a single convolution kernel. (Author‐created schematic virtual face image; not derived from any real‐person photograph or database.) Each image extracts 32 × 32 patches from the recording area and is convolved with a single convolution kernel. The resulting scalar feature value is compared with a fixed threshold to classify the image as “Face” or “Non‐face”. The score distribution illustrates how true positives (TP), true negatives (TN), false positives (FP), and false negatives (FN) are determined. (c) Mapped photoresponsivity matrix of the 32 × 32 face detection kernel. The photoresponsivity (from −1000^−^ to + 1000 mA W^−1^) of the device was mapped to the originally trained kernel weights. (d) Distributions of the kernel weights before (top) and after (bottom) mapping to device photoresponsivity. (e) Confusion matrices using the mapped photoresponsivity‐based kernel for 2064 patches. At threshold  =  0, the system achieves balanced performance (Accuracy: 87.79%; Precision: 0.8903; Recall: 0.8628; F1‐score: 0.8763). False positive errors are corrected by the powerful DNN which is triggered ON by the detection. (f) Distributions of classifier scores obtained from convolving face and non‐face samples with the photoresponsivity‐mapped kernel. The vertical dashed lines represent decision thresholds at 0 (black) and −1.525 (blue). (g) Quantitative comparison of classification metrics (Accuracy, Precision, Recall, F1‐Score) using the original kernel and the photoresponsivity‐mapped kernel. (h) Receiver operating characteristic (ROC) curves comparing the original kernel and measured kernels with write noise.

In many applications with real‐time processing requirements such as drone‐based human search and IoT vision nodes for human occupancy or pedestrian detection, much of the energy is expended in processing redundant image regions [52, 53]. Biological vision systems handle this by separate pathways that estimate where the salient objects are located and what their identities are [54]. Adopting such saliency‐based filtering can significantly reduce the downstream computational burden and energy consumption in artificial systems as well. As shown in Figure 6a, a UAV performs surveillance at a road intersection. The aerial camera captures high‐resolution images, which are segmented into multiple small patches (Figure 6b). Rather than using multi‐layer neural networks acting on all the patches, our in‐sensor array can serve as an intelligent front‐end saliency filter with only a single layer to efficiently discriminate between potential “face” and “non‐face” regions. As illustrated, only the face‐containing patches corresponding to actual pedestrians are passed to the next stage (e.g. high‐precision DNN) for confirmation of face with higher precision, identity recognition or behavior analysis, while most of the background information is discarded. This design substantially reduces the amount of data transmitted and processed downstream, which is particularly advantageous in resource‐constrained edge devices such as UAVs.

To achieve this, the face detection model was trained with inspiration from cascade classifiers with the first stage implemented entirely in‐sensor. However, unlike Haar classifiers used earlier, we use modern DNNs trained by backpropagation as the rest of the classifier that gets triggered ON by the detections in the first stage [55]. Our system was trained and evaluated using the FDDB (Face Detection Data Set and Benchmark), which provides a realistic dataset of unconstrained faces in natural scenes. We segmented all images of the dataset into 32 × 32 pixels and obtained 146 368 patches in total. Then, these patches are convolved with a trained convolutional 32 × 32 kernel. The scalar features (confidence score) obtained from the convolution operation represent the response strength, which can be compared with a fixed threshold, and a binary decision will be produced: “Face” or “Non‐face”. Here, the decision threshold defines the operating regime of the front‐end detector. A threshold of 0 corresponds to a natural decision boundary where positive kernel responses indicate stronger correlation with face‐like patterns, while negative responses correspond to non‐face regions. In addition, a lower threshold (−1.525) is introduced to illustrate a high‐recall operating regime, which is desirable for cascade‐style front‐end filtering where missed detections should be minimized, and false positives can be further rejected by downstream processing. These two thresholds, therefore represent two typical trade‐offs between computational reduction and detection completeness, and the exact threshold can be adjusted depending on the requirements of specific applications. When the threshold is 0(−1.525), only 22.2% (54.4%) of the windows are judged as “Face” that need to be processed for downstream tasks. While discarding 77.8% (45.6%) “Non‐face” patches, which enables over 4.51× (1.84×) reduction in subsequent computation. This ratio is expected to be even higher for real‐world surveillance or traffic monitoring datasets, where non‐face regions dominate. Details of the binary classification results, such as precision, accuracy, recall, etc. are reported in Note S1.

To translate the trained kernel into in‐sensor physical weights, we mapped the 32 × 32 weight matrix (Figure S26) into the measurable photoresponsivity of our array. As shown in Figure 6c, each weight (range from −0.79 to +0.52) was linearly mapped into a photoresponsivity value ranging from −1000 to 1000 mA·W^−^^1^, ensuring compatibility with the programmable range of the device. The mapped kernel closely reproduces the original kernel, such as the bright eye and mouth regions, without visible distortion. Figure 6d further supports this by comparing the distribution of original weights and mapped values‐ the closest photoresponsivity value to the mapped weight was used in the actual programming process. The mapping introduces a negligible shift in distribution, enabling direct translation of trained weights into physically meaningful device parameters. To evaluate classification performance exactly, we randomly selected 2064 samples from the dataset (1035 face samples and 1029 non‐face samples) that enable controlled comparison across multiple parameters to construct confusion matrices. As shown in Figure S27a, we first used the original 32 × 32 kernel for simulation. When the threshold is 0, the model achieved strong performance (Accuracy: 88.28%, Precision: 0.9025, Recall: 0.8589, F1‐score: 0.8802). The “Recall” is critical in face detection scenarios to minimize missed detections, and it can be further enhanced by setting a lower threshold of −1.525. This setting increases the “Recall” to 0.9517, though at the expense of reduced precision, a common trade‐off in cascade classification systems since False positives get corrected by the following strong classifier.

To validate the usability of the actual weights, we also performed the same test using the photoresponsivity‐mapped weights (Accuracy: 87.79%; Precision: 0.8903; Recall: 0.8628; F1‐score: 0.8763) (Figure S27b; Figure 6e). Both thresholds yielded confusion matrices highly consistent with those of the original kernel, which confirms that the in‐sensor operation can be performed directly on programmable device arrays. To further understand the statistical behavior of the system, we analyzed the distribution of output classifier scores. As shown in Figure 6f, after convolving with the photoresponsivity‐mapped kernel of face and non‐face inputs, the scores are plotted for categories. The distributions reveal a separation between the two classes, allowing for effective classification via thresholding, like the convolutional output score distribution using the original kernel (Figure S28). Two thresholds are highlighted: the default 0, and a lower threshold −1.525 that enhances recall at the cost of precision. This highlights an important advantage of this system, the post‐fabrication tunability, enabling the classification behavior to be adapted for different application requirements (e.g., prioritizing detection vs. rejection). As shown in Figure 6g, the accuracy, precision, recall, and F1‐score can be adjusted by threshold tuning, both for the original and mapped kernels. To assess the classifier robustness to write noise, the same kernel was programmed five times (measured kernel 2–6), and the ROC curves are plotted for the different cases in Figure 6h. These five cases simulate the effect of potential device noise or fabrication variability (Note S2). All the kernels show nearly identical ROC performance and close AUC (Area Under the Curve) value, confirming the reliability and robustness of the proposed device‐mapped design for real‐world classification tasks.

## 3 Conclusion

We have developed a scalable perovskite‐based platform for in‐sensor computing, enabling reconfigurable photoresponsivity modulation through control of electric fields. By incorporating EFC into halide perovskite thin films, our system achieves bidirectionally tunable photoresponsivity up to ∼1000 distinguishable states, with a record‐high dynamic range of ±1120 mA W^−^^1^. Consistent device performance was maintained over 30 days of ambient storage and under thermal cycling up to 80°C. Leveraging the performance, we demonstrated key vision tasks including optical convolution and high‐accuracy image classification. The superior processability and scalability of this system enable single‐layer filtering with a 32 × 32 array for face detection, achieving 95.2% sensitivity with only 22.2% cases activated for downstream tasks. These results verify the feasibility of integrating sensing and computing in an energy‐efficient architecture, offering a practical route toward large‐area, cost‐effective integration.

## 4 Experimental Section/Methods

### 4.1 Preparation Precursor Solution

To synthesize the nominal Cs_0.05_(MA_0.16_FA_0.84_)_0.95_Pb(I_0.83_Br_0.17_)_3_ perovskite, a precursor solution was prepared using a molar ratio corresponding to the stoichiometric formula. Cesium iodide (CsI, 99.99% trace metals basis, Sigma–Aldrich) 16.9 mg, formamidinium iodide (FAI, 99.99% trace elements basis, GreatCell Solar) 178.4 mg, and methylammonium bromide (MABr, 99.99%, GreatCell Solar) 22.1 mg were weighed and dissolved together with lead(II) iodide (PbI_2_, 99.99% trace metals basis, Sigma–Aldrich) 492.3 mg and lead(II) bromide (PbBr_2_, 99.99% trace metals basis, Sigma–Aldrich) 85.4 mg in a co‐solvent mixture of N, N‐dimethylformamide (DMF) and dimethyl sulfoxide (DMSO) at a volume ratio of 4:1 (DMF: anhydrous 98.8%, 800 µL, DMSO: anhydrous ≥99.9%, 200 µL, both from Sigma–Aldrich). The final perovskite precursor solution had a molar composition of 1.3 M Cs_0.05_(MA_0.16_FA_0.84_)_0.95_Pb(I_0.83_Br_0.17_)_3_. Additionally, 0.5 mg/mL EFC [poly (vinylidene fluoride‐trifluoroethylene), P(VDF‐TrFE), Sigma–Aldrich] was incorporated into the perovskite precursor solution and stirred at 60°C for 8 h to ensure thorough dissolution and homogeneity. The resulting solution was filtered through a 0.22 µm hydrophobic polytetrafluoroethylene (PTFE) filter to remove any undissolved particles before use.

### 4.2 Devices Fabrication

First, the Si/SiO_2_ substrate (300 nm thermally grown silicon oxide on Si wafer) was ultrasonically cleaned in a 2% (v/v) Decon 90 solution dispersed in deionized (DI) water for 15 min. This was followed by sequential ultrasonic cleaning in acetone and isopropanol (IPA), each for 15 min. The cleaned substrate was blown dry using nitrogen gas (N_2_) and baked at 80°C for 30 min in a drying oven to remove residual solvents. Next, a positive photoresist (AZ 5214E, MicroChemicals GmbH) was spin‐coated at 4000 rpm (acceleration: 1000 rpm/s) for 30 s and soft‐baked at 115°C for 2 min. The electrode pattern was then defined by ultraviolet (UV) exposure through a pre‐designed mask using a contact photolithography mask aligner (Karl Suss MJB 4, SÜSS MicroTec). The exposed pattern was developed for 50 s in an AZ developer solution (AZ 400K, diluted 1:3 with DI water), followed by rinsing with DI water. Subsequently, 5 nm chromium (Cr) and 45 nm gold (Au) layers were deposited using electron beam evaporation at a deposition rate of 0.1 Å/s under high vacuum (∼10^−^^6^ mbar). A lift‐off process was performed by immersing the sample in acetone with gentle ultrasonication for 5 min, followed by rinsing with IPA for 1 min, yielding the final electrode array. For perovskite deposition, the perovskite precursor solution 60 µL (as described in Section preparation precursor solution) was dispensed onto the patterned substrate, followed by a spin‐coating process: 1000 rpm for 10 s (acceleration: 200 rpm/s), and 6000 rpm for 20 s (acceleration: 1000 rpm/s). Chlorobenzene (anti‐solvent, 120 µL) was dropped onto the spinning substrate 10 s before the end of the second step. The coated substrates were immediately transferred to a hot plate and annealed at 100°C for 1 h to promote crystallization.

### 4.3 Device Measurements

Electrical measurements were performed using a continuous‐pump vacuum probe station (Lakeshore) equipped with temperature control capabilities for measurements under variable thermal conditions. A Keithley 4200‐SCS semiconductor parameter analyzer was used to apply either a swept voltage bias or a constant voltage bias, while the Keysight B2912B precision source/measure unit (SMU) was employed to characterize response speed and retention characteristics. Photoresponse measurements were conducted under illumination from a tunable LED light source (ThorLabs), with tunable intensity and wavelength. The emitted light covered three spectral bands: red (λ = 623 nm), green (λ = 525 nm), and blue (λ = 445 nm). The incident light was directed vertically onto the device through a quartz optical window integrated into the probe station, ensuring uniform illumination. The light intensity was systematically varied to study the light‐dependent electrical characteristics.

### 4.4 Characterization

Scanning electron microscopy (SEM) images of the perovskite films were acquired using a field‐emission scanning electron microscope (FE‐SEM, JEOL JSM‐6340F) operated at an accelerating voltage of 5.0 kV. The surface roughness, contact potential difference (CPD), and ferroelectricity of the films were characterized using an atomic force microscope (AFM, Cypher S, Asylum Research) in multiple operation modes: tapping mode for topography, scanning Kelvin probe microscopy (SKPM) for surface potential, and piezoresponse microscopy (PFM) for ferroelectric phase imaging (In‐plane polarization mode). All AFM measurements were performed under ambient conditions.

### 4.5 Dataset

For the convolution operation, grayscale images were preprocessed and normalized using Python‐based image processing libraries (NumPy). In the MNIST digit recognition task, the whole 60 000 training images were used for model training, while the entire 10 000‐image dataset was used for evaluation and testing. For the face detection task, 146 368 patches were segmented from the dataset of Face Detection Dataset and Benchmark (FDDB), all of which were used for the computational efficiency task. All face annotations were cropped and resized into 32 × 32 grayscale patches. Both the training and test datasets were constructed and processed using Python.

## Conflicts of Interest

The authors declare no conflicts of interest.

## Supporting information




**Supporting File**: adma73243‐sup‐0001‐SuppMat.docx.

## Data Availability

The data that support the findings of this study are available from the corresponding authors upon reasonable request.
