# Supplementary material for: A Scalable Perovskite Platform With Multi‐State Photoresponsivity for In‐Sensor Saliency Detection
Source: Adv Mater. 2026 Apr 29;38(32):e73243. doi: 10.1002/adma.73243 (PMC13244815; doi:10.1002/adma.73243)
Supplement: Supplementary file 1 — Supporting File: adma73243‐sup‐0001‐SuppMat.docx. [file ADMA-38-e73243-s001.docx]

**A Scalable Perovskite Platform with Multi-state Photoresponsivity for In-Sensor Saliency Detection**

Xuechao Xing^1^, Anubhab Tripathi^2^, Si En Ng^1^, Natalia Yantara^3^, Yue Gong^4^, Qinjie Wu^1^, Yeow Boon Tay^1^, Wen Siang Lew^5^, Yang Chai^6^, Arindam Basu^2^* & Nripan Mathews^1,3^*

^1^School of Materials Science and Engineering, Nanyang Technological University, 50 Nanyang Avenue, 639798, Singapore

^2^Department of Electrical Engineering, City University of Hong Kong, YEUNG-G6410, 999077, Hong Kong

^3^Energy Research Institute @ NTU (ERI@N), Nanyang Technological University, 50 Nanyang Drive, 637553, Singapore

^4^Interdisciplinary Graduate School, Nanyang Technological University, 50 Nanyang Drive, 639798, Singapore

^5^School of Physical and Mathematical Sciences, Nanyang Technological University, 21 Nanyang Link, 637371, Singapore

^6^Department of Applied Physics, The Hong Kong Polytechnic University, 11 Yuk Choi Rd, Hung Hom,999077, Hong Kong

**Table of Contents**

Table S1 | Device fabrication comparison of in-sensor computing reporting devices.

Table S2 | Benchmark the performance of the perovskite-EFC photovoltaic devices against other recent state-of-the-art works.

Figure S1 | The processibility and scalability of 32 × 32 array.

Figure S2 | Relaxation behavior of the I_sc_ in the Au/CsFAMA/Au device.

Figure S3 | I–V curves of Au/CsFAMA/Au devices under different programming electric fields.

Figure S4| Influence of EFC concentration on the optical properties and morphology of CsFAMA perovskite films.

Figure S5 | Thermal cycling stability evaluation of control and target devices.

Figure S6 | I–V curves of Au/EFC/Au devices under different programming electric fields.

Figure S7 | Schematic diagram of grain size analysis of perovskite samples.

Figure S8 | AFM analysis of surface roughness for the control and target perovskite films.

Figure S9 | KPFM surface potential map acquired over a 10 μm × 10 μm region within the control film after lateral electric-field programming.

Figure S10 | Time-dependent KPFM surface potential evolution within a localized 5 μm × 5 μm scan window before (Initial) and after (0, 5, 10, and 30 min) lateral electrical programming.

Figure S11 | Retention characteristics of the control and target devices (read @ 0 V).

Figure S12 | In-plane piezoresponse force microscopy (PFM) characterization of the control CsFAMA sample.

Figure S13 | Structural integrity analysis via Grazing-Incidence Wide-Angle X-ray Scattering (GIWAXS)

Figure S14 | The dark current of both the control and target samples.

Figure S15 | Retention and stability of programmed photoresponsivity states.

Figure S16 | Analysis of photoresponsivity under high-intensity illumination and photoresponsivity states.

Figure S17 | Analysis of photoresponsivity controllability in the control sample.

Figure S18 | Analysis of photoresponsivity controllability in the target sample.

Figure S19 | Multi-wavelength photoresponse characteristics of the target sample.

Figure S20 | Demonstrating substrate compatibility and device uniformity.

Figure S21 | Evaluation of mechanical bending effects on the flexible device performance.

Figure S22 | Evaluation of the initial state of the array devices.

Figure S23 | Image denoising using a Gaussian convolution kernel.

Figure S24 | Evaluate the denoising effect of the Gaussian kernel-based CNN system.

Figure S25 | Confusion matrix of the MNIST classification task using software simulation.

Figure S26 | The 32×32 confusion matrix of a single-stage classifier for face detection.

Figure S27 | Confusion matrices using the original and mapped kernel for 2064 patches with different thresholds.

Figure S28 | The distribution of scores using the original kernel.

Supplementary Note 1| Evaluation Metrics for Binary Classification.

Supplementary Note 2 | ROC Curve and AUC.

**Table S1.** **Device fabrication comparison of in-sensor computing reporting devices.**

| **Materials** | **Device fabrication** | **Substrate** | **Array size** | **Device-to-device variability** |
| --- | --- | --- | --- | --- |
| WS_2_ nanotubes **^1^** | Disperse | 500 nm-SiO_2_/Si | 4×4 | / |
| MoS_2-x_ flake (pretreatment) **^2^** | Mechanically exfoliate and transfer | SiO_2_–p^++^Si | / | / |
| MoTe_2_ flake/P(VDF-TrFE) **^3^** | Mechanically exfoliate and transfer | 280 nm-SiO_2_/Si | 3×9 | 27 devices |
| 3R-WS_2_ flake **^4^** | Mechanically exfoliate and transfer | SiO_2_/Si | / | / |
| WSe_2_/Al_2_O_3_/HfO_2_/Al_2_O_3_ **^5^** | Mechanically exfoliate and transfer | 300 nm-SiO_2_/Si | 3×3 | / |
| SWNT@GDY **^6^** | Aerosol Jet printing | 300 nm-SiO_2_/Si | 3×9 | 27 devices |
| WS_2_ flake **^7^** | Mechanically exfoliate and transfer | 280 nm-SiO_2_/Si | 3×9 | 27 devices |
| Pb(Zr_0.2_Ti_0.8_)O_3_(PZT) **^8^** | Epitaxial growth | (001) STO single crystalline | 2 × 2 | 4 devices |
| Pb(Zr_0.2_Ti_0.8_)O_3_(PZT) **^9^** | Epitaxial growth | (001) STO single crystalline | / | / |
| Black phosphorus **^10^** | Mechanically exfoliate and transfer | 300 nm-SiO_2_/Si | 3×4 | 9 devices |
| PdSe_2_/ MoTe_2_ **^11^** | Mechanically exfoliate and transfer | 300 nm-SiO_2_/Si | / | / |
| MoS_2-x_O_x_ nanocrystals **^12^** | Humidifier spray | SiO_2_/Si | 3×3 | / |
| MaPbI_3_/Bi_2_O_2_Se **^13^** | Spin coat + Mechanically exfoliate and transfer | HfO_2_/Si | 3×3 | 9 devices |
| **This work** | **Spin coat** | 300 nm-SiO_2_/Si and **PET** | **32×32** | **64 areas** |


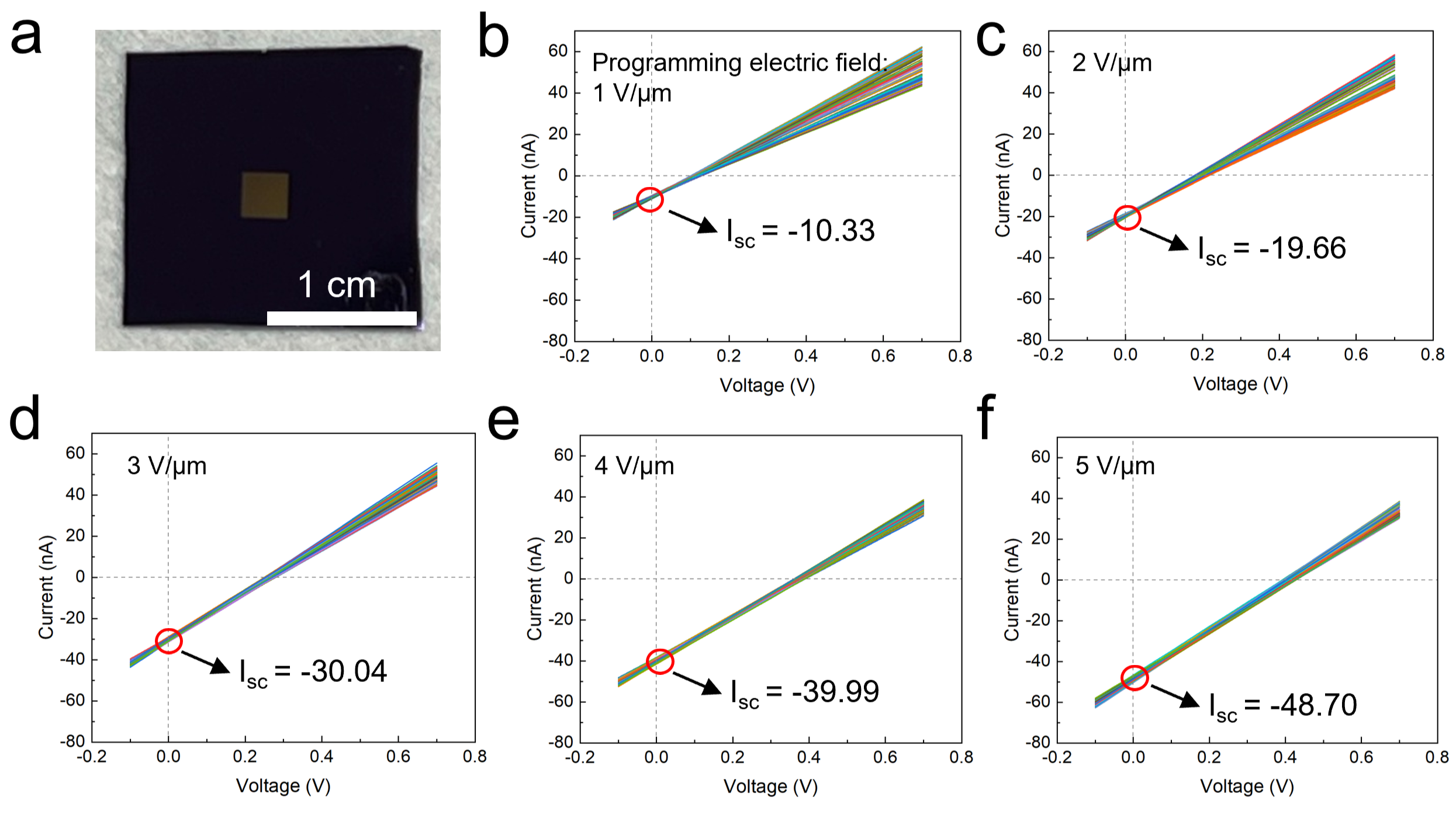


**Figure S1|** **The processibility and scalability of 32 × 32 array.**

**a.** Optical images of 1024 devices on a 3.2 mm × 3.2mm area.

**b-f.** The I_sc_ of each sampled device was measured after electric field programming from 1 V/μm to 5 V/μm.

The results demonstrate the uniformity and low variability of the perovskite-based controllable photovoltaic devices across the array.


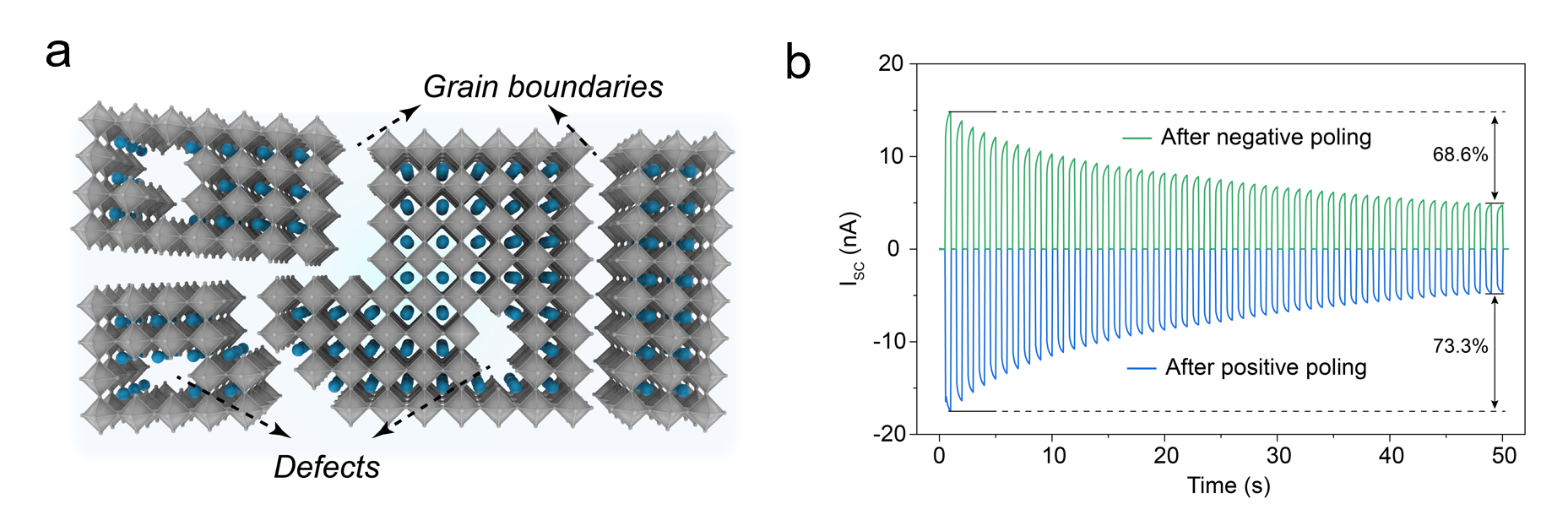


**Figure S2|** **Relaxation behavior of the I_sc_ in the Au/CsFAMA/Au device**.

**a.** The lattice structure of perovskite. The defects and grain boundaries act as pathways for ion migration.

**b.** After polarization by positive and negative electric fields, the I_sc_ exhibits a rapid decay within one minute, decreasing by approximately 70% relative to its initial value.

The rapid relaxation of the I_sc_ is governed by the ion migration dynamics of the material. After the removal of the external electric field, field-driven ions or vacancies tend to redistribute back toward their initial equilibrium state, making it difficult to maintain the electrically induced state over an extended period.


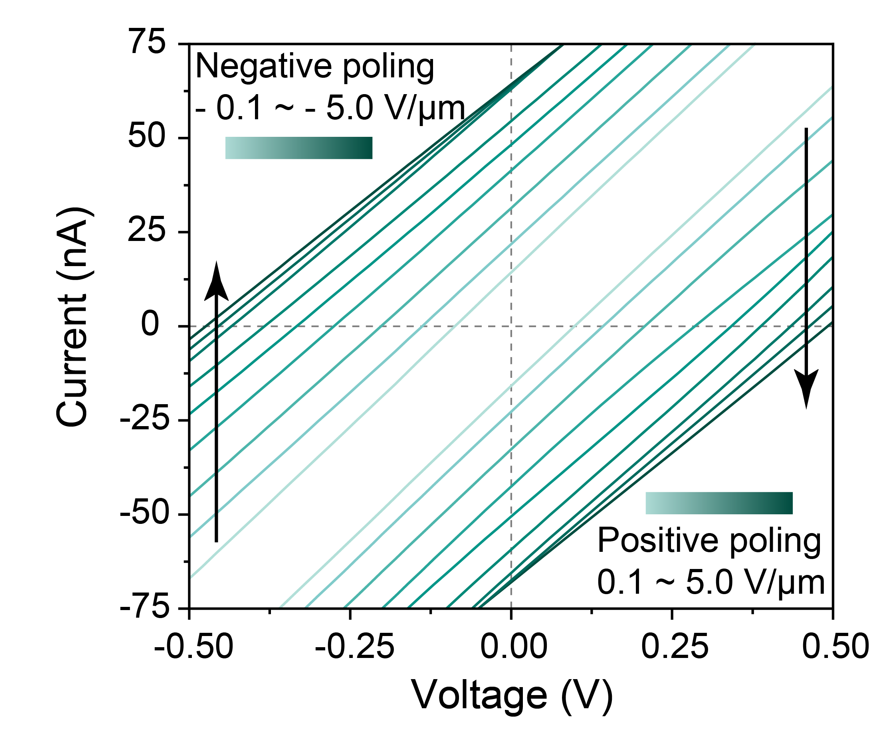


**Figure S3|** ***I–V* curves of Au/CsFAMA/Au devices under different programming electric fields.** The results demonstrate the switchable photovoltaic characteristics of the control device. sweeping rate of 0.05 V/step, illumination intensity:50 mW cm^-2^, light wavelength: 623 nm.

Notably, effective photovoltaic performance (V_oc_ and I_sc_) is observed at a relatively low programming field of 0.1 V/μm, indicating that ions within the perovskite film migrate readily under mild electric field conditions.


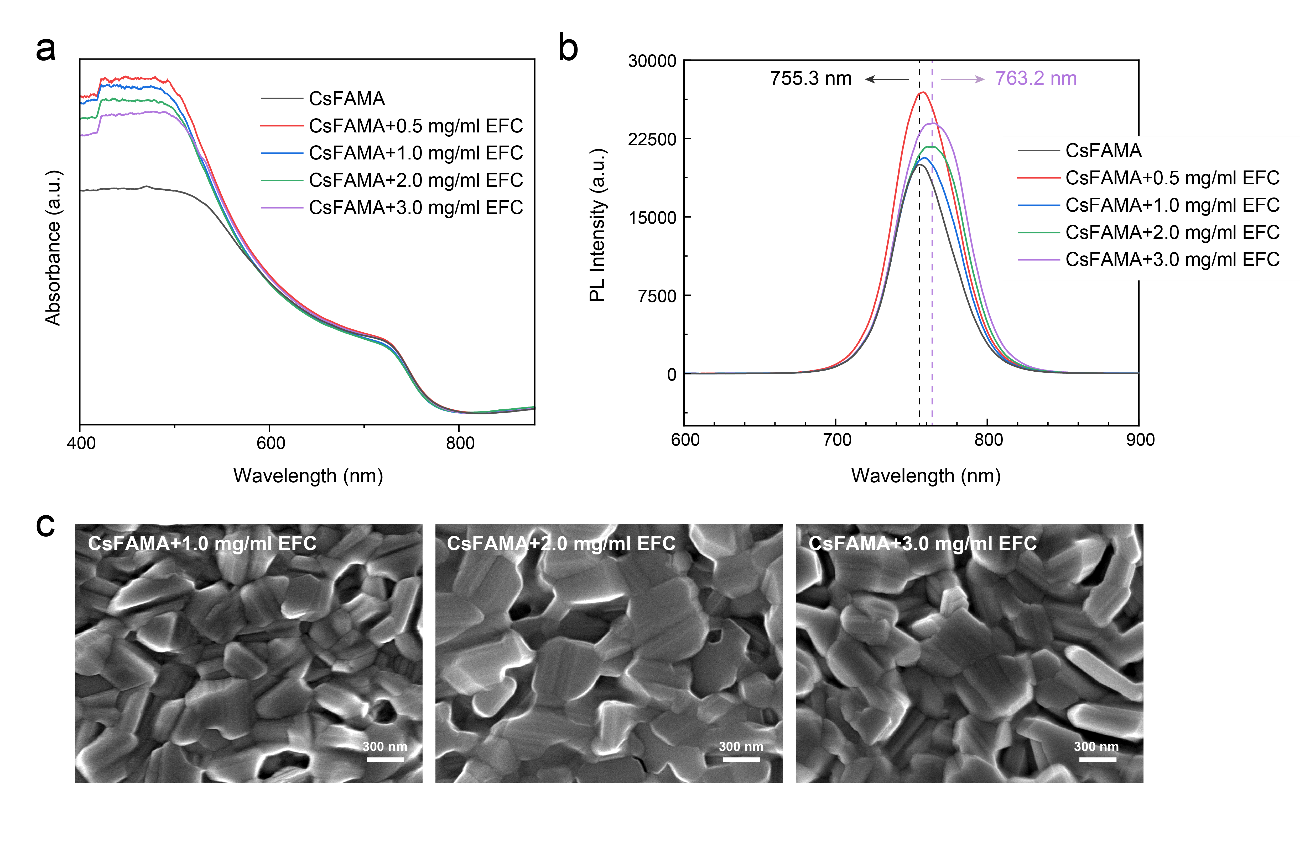


**Figure S4| Influence of EFC concentration on the optical properties and morphology of CsFAMA perovskite films.**

**a**. Absorbance spectra of CsFAMA thin films of different EFC concentrations (0-3 mg mL^-1^). **b**. Steady-state photoluminescence (PL) spectra of the corresponding films. The film containing 0.5 mg mL^-1^ EFC exhibits the strongest emission intensity, suggesting improved film quality at moderate polymer loading. c. SEM images of CsFAMA thin films of different EFC concentrations (1, 2, and 3 mg mL^-1^), Scale bar: 300 nm.


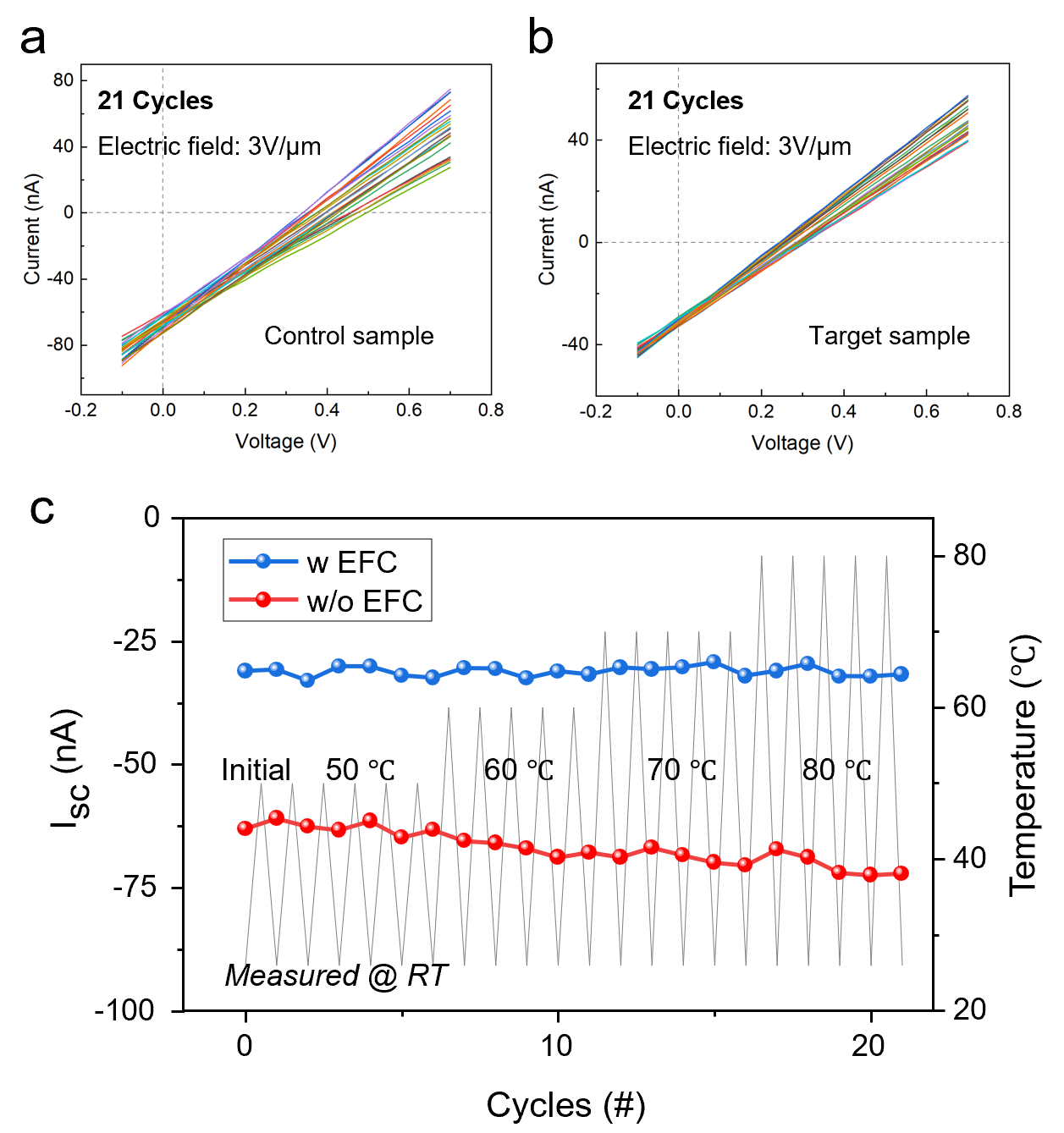


**Figure S5| Thermal cycling stability evaluation of control and target devices.**

**a.** The I_sc_ of control sample and **b.** the target sample were tested for five cycles at different temperatures. Thermal cycling was performed using a pre-set hot plate, with each cycle consisting of 15 minutes of heating. A total of five cycles were conducted at each temperature step, ranging from 50 °C to 80 °C, to assess the thermal reliability and stability of the devices under repeated thermal stress.

**c.** I_sc_ variations were recorded for both the control and target devices at room temperature and elevated temperature (+ 80 °C). The results reveal that the I_sc_ of the control device increases with rising temperature, whereas the target device maintains a stable I_sc_ across the entire temperature range, demonstrating thermal stability and robustness.


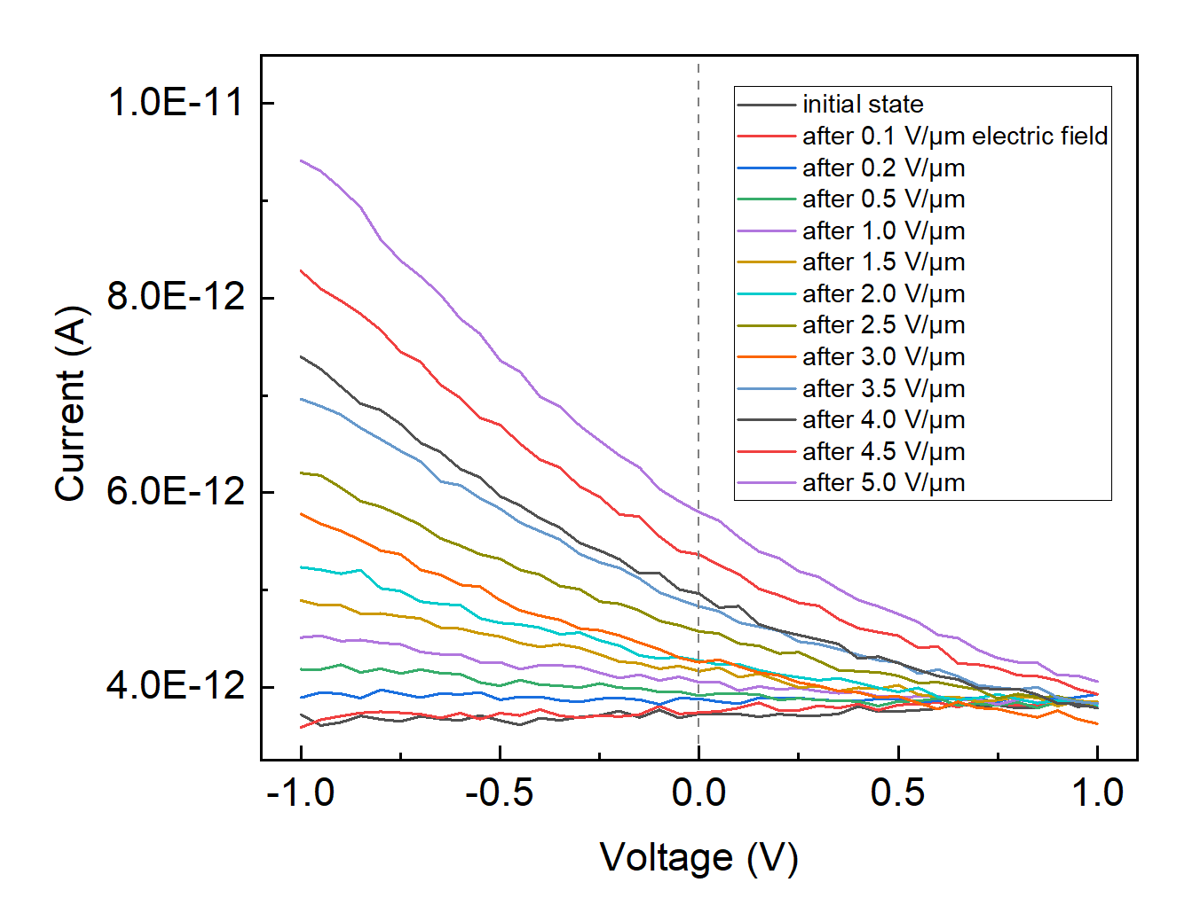


**Figure S6|** ***I–V* curves of Au/EFC/Au devices under different programming electric fields.**

After electric field programming, the sample exhibits a slightly photocurrent response; The overall current remains in the picoampere to tens of picoamperes range, indicating that the device is in a low-conductivity state. Sweeping rate of 0.05 V/step, illumination intensity:50 mW cm^-2^, light wavelength: 623 nm.

This result confirms that EFC alone does not generate a photovoltaic effect. Instead, the photovoltaic behavior originates from the perovskite film, while EFC functions as a modulating component, influencing ion migration or interface properties.


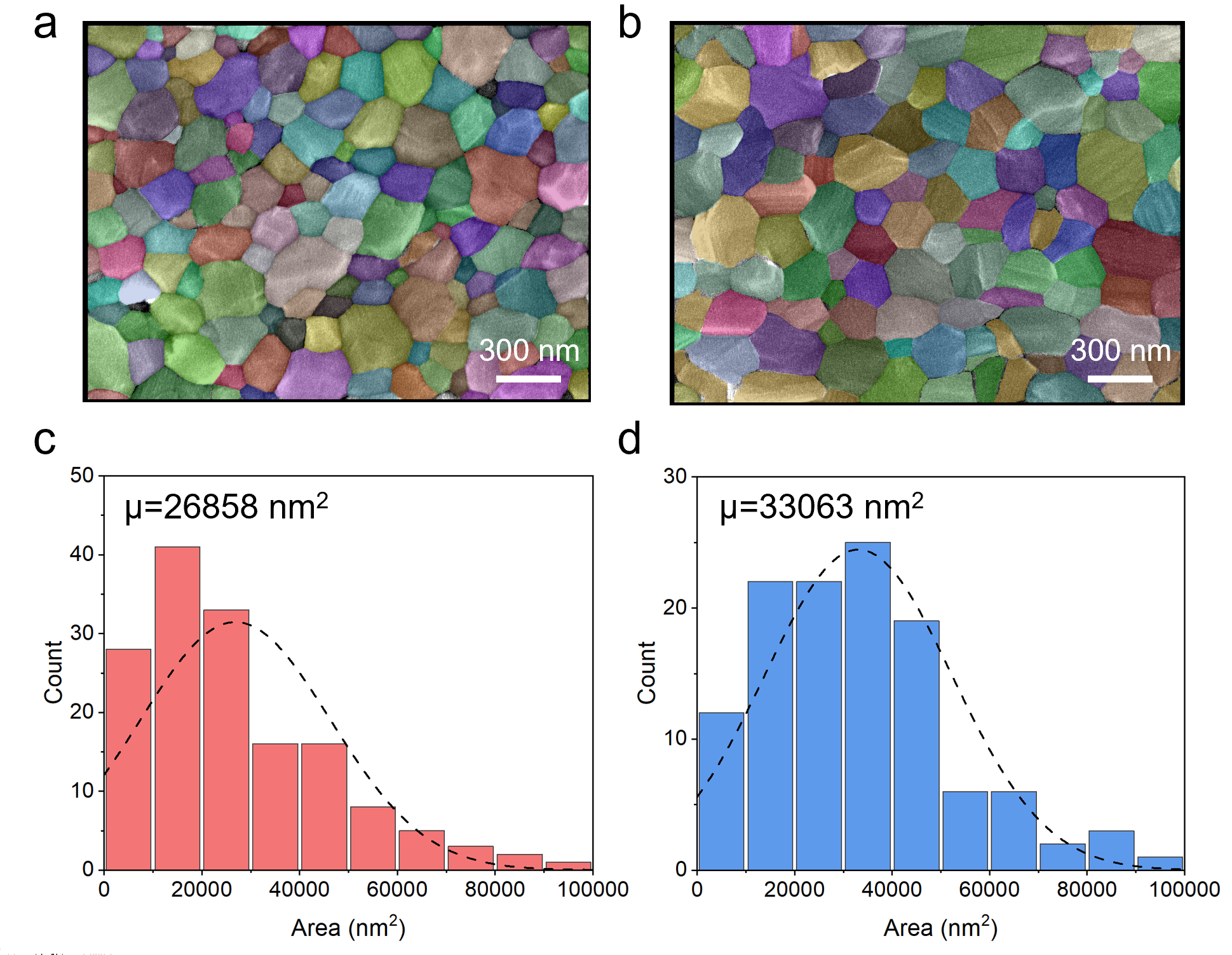


**Figure S7| Schematic diagram of grain size analysis of perovskite samples.**

**a.** Color-mapped grain differentiation of the perovskite film in the control sample and **b.** in the target sample.

**c.** The average area statistics of all grains in the scanning area of the control sample and **d.** the target sample.

Within the same scanned area, the control sample contains 153 individual grains, whereas the target sample contains only 118 grains. This reduction in grain number suggests that EFC was successfully introduced and exerts a noticeable influence on the grain growth and grain boundary characteristics of the perovskite film.


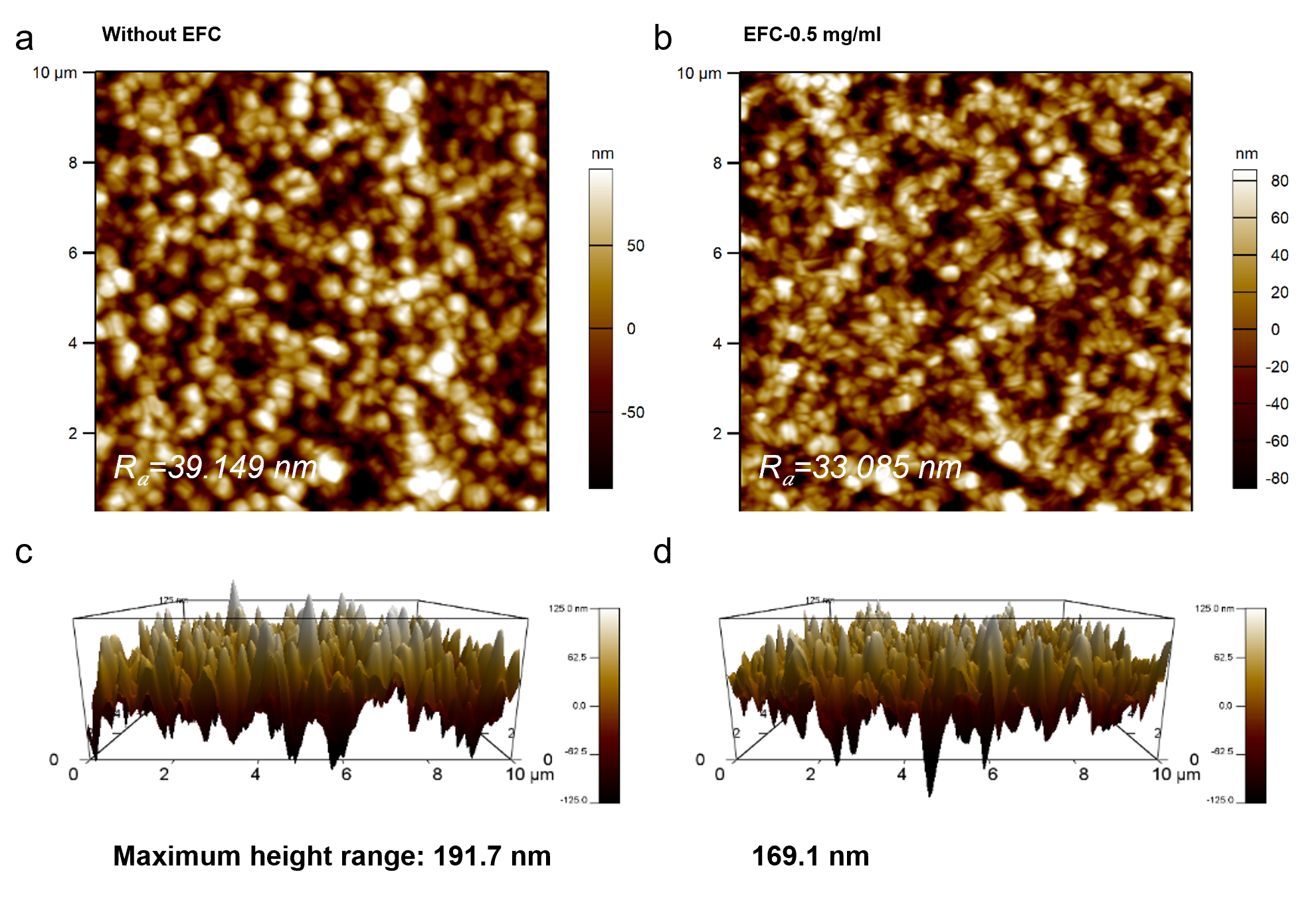


**Figure S8| AFM analysis of surface roughness for the control and target perovskite films.**

**a.** Surface roughness of the pristine CsFAMA film (control sample), with an average roughness (Ra) of 39.149 nm.

**b.** Surface roughness of the perovskite film mixed with EFC (target sample), showing a reduced Ra of 33.085 nm.

**c, d.** Corresponding 3D height maps of the control (c) and target (d) samples, providing a visual representation of surface topography and roughness.

Adev [Ra] is commonly used to describe roughness.

Average difference of n data. *Z* is the height information


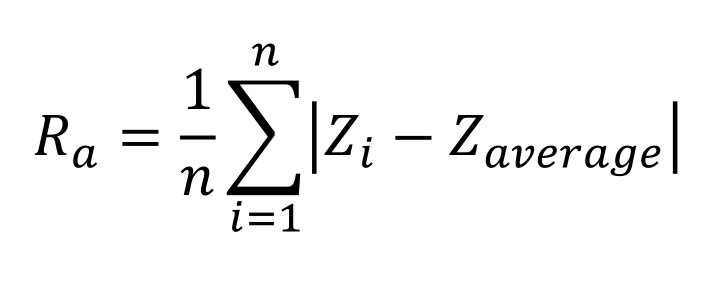


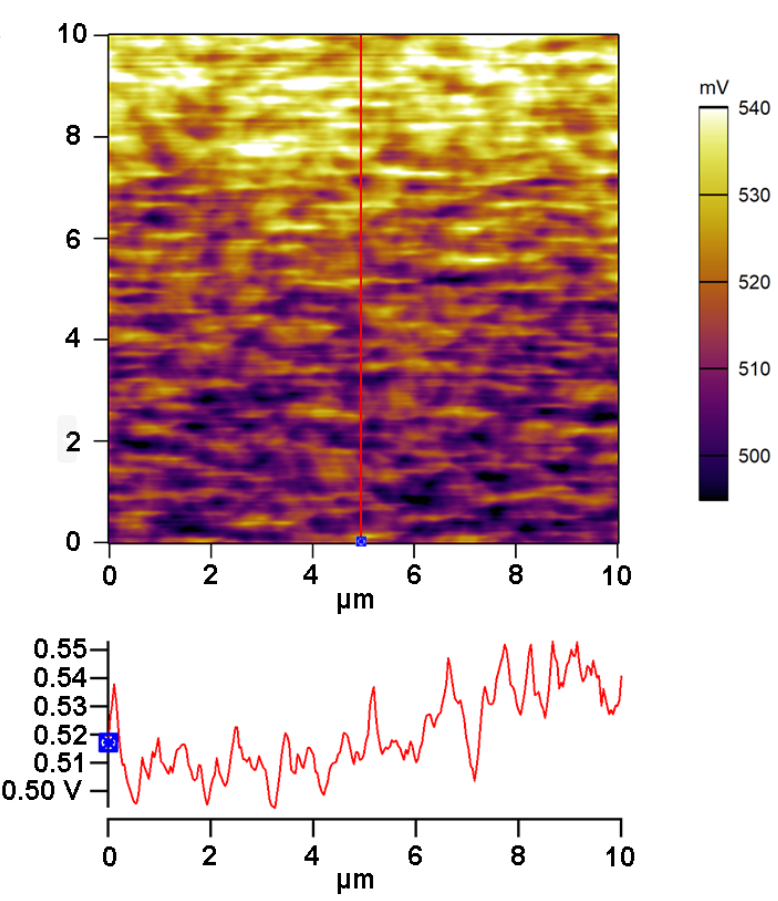


**Figure S9| KPFM surface potential map acquired over a 10 μm × 10 μm region within the control film after lateral electric-field programming.** A clear horizontal potential gradient is observed across the scan area, as confirmed by the corresponding line profile (bottom), indicating the presence of a lateral internal potential gradient induced by ionic redistribution.


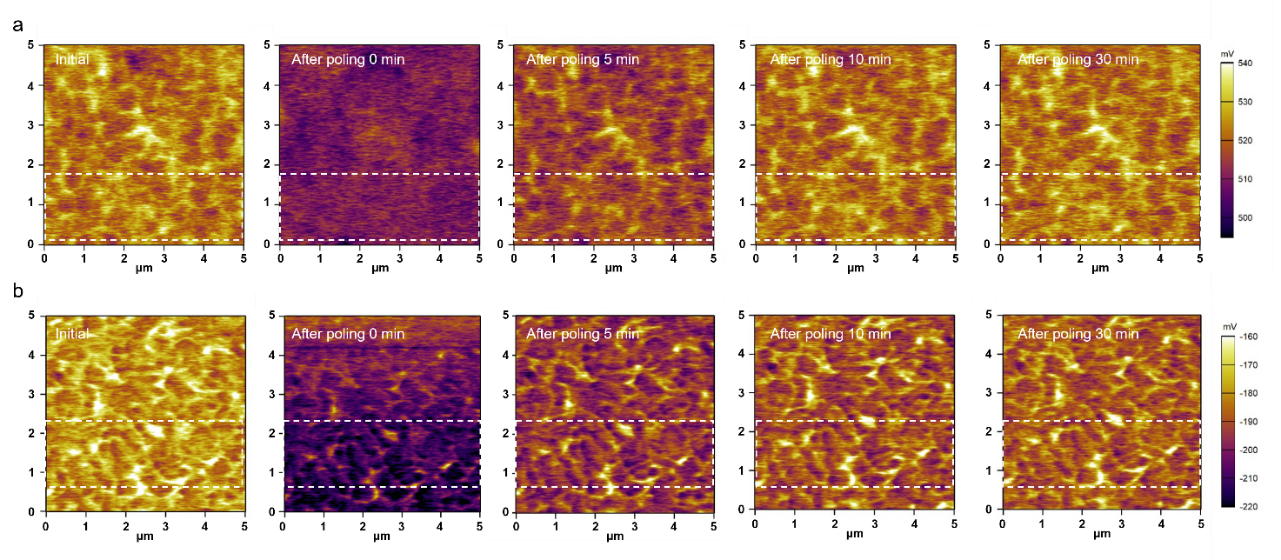


**Figure S10| Time-dependent KPFM surface potential evolution within a localized 5 μm × 5 μm scan window before (Initial) and after (0, 5, 10, and 30 min) lateral electrical programming.**

**a.** Top row: Control perovskite film. **b.** EFC-incorporated target film. The programming field was applied laterally along the electrode gap direction. The dashed white boxes (~5 μm × 1.8 μm) indicate the representative subregions used for quantitative CPD analysis, which displayed in Figure 2i–j.


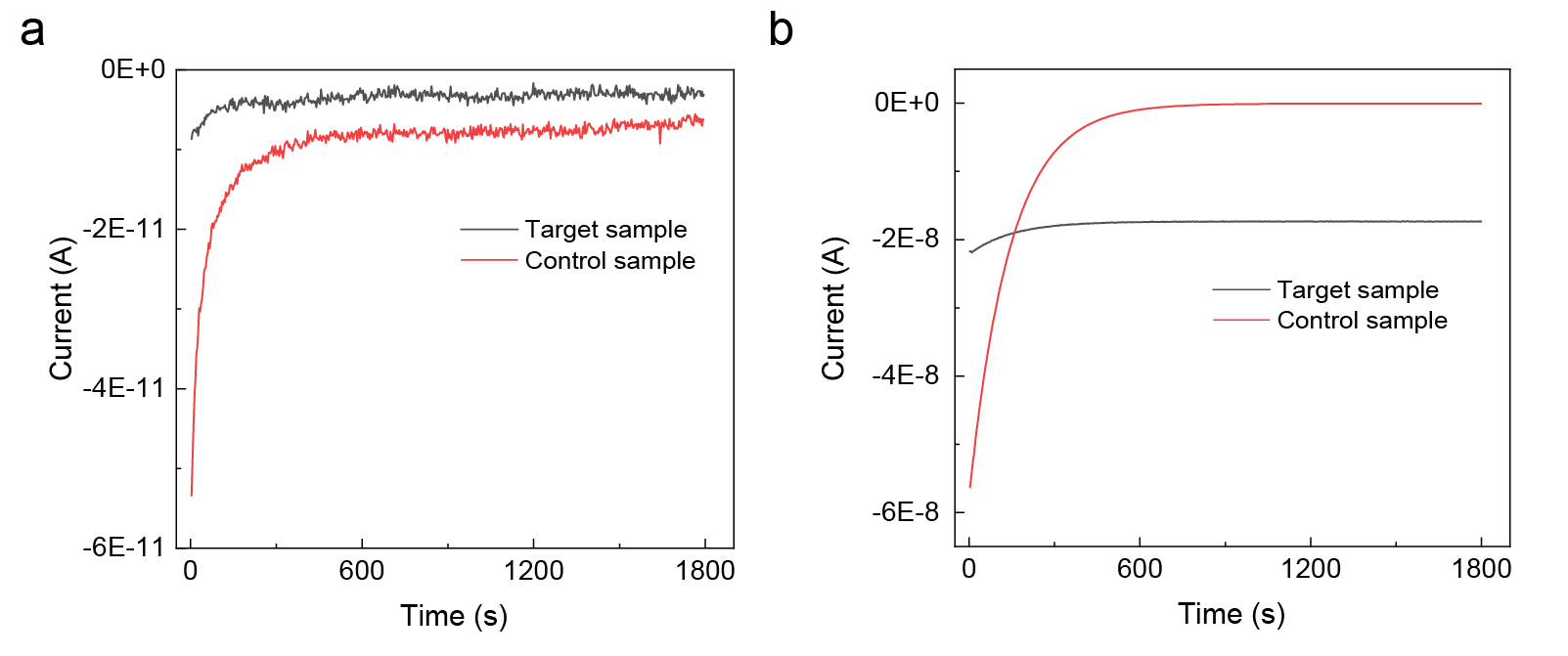


**Figure S11| Retention characteristics of the control and target devices (read @ 0 V).**

**a.** Dark current of the control and target samples recorded for 1800 s under dark conditions. The control sample shows a much larger current variation, indicating faster relaxation of the electrically redistributed ionic configuration, whereas the target sample exhibits a smaller dark current and much weaker temporal evolution. **b.** Photocurrent (I_sc_) of the control and target samples measured at 50 mW cm^-2^. Compared with the control sample, the target sample maintains a much more stable photocurrent after programming, demonstrating improved retention of the programmed photovoltaic state.


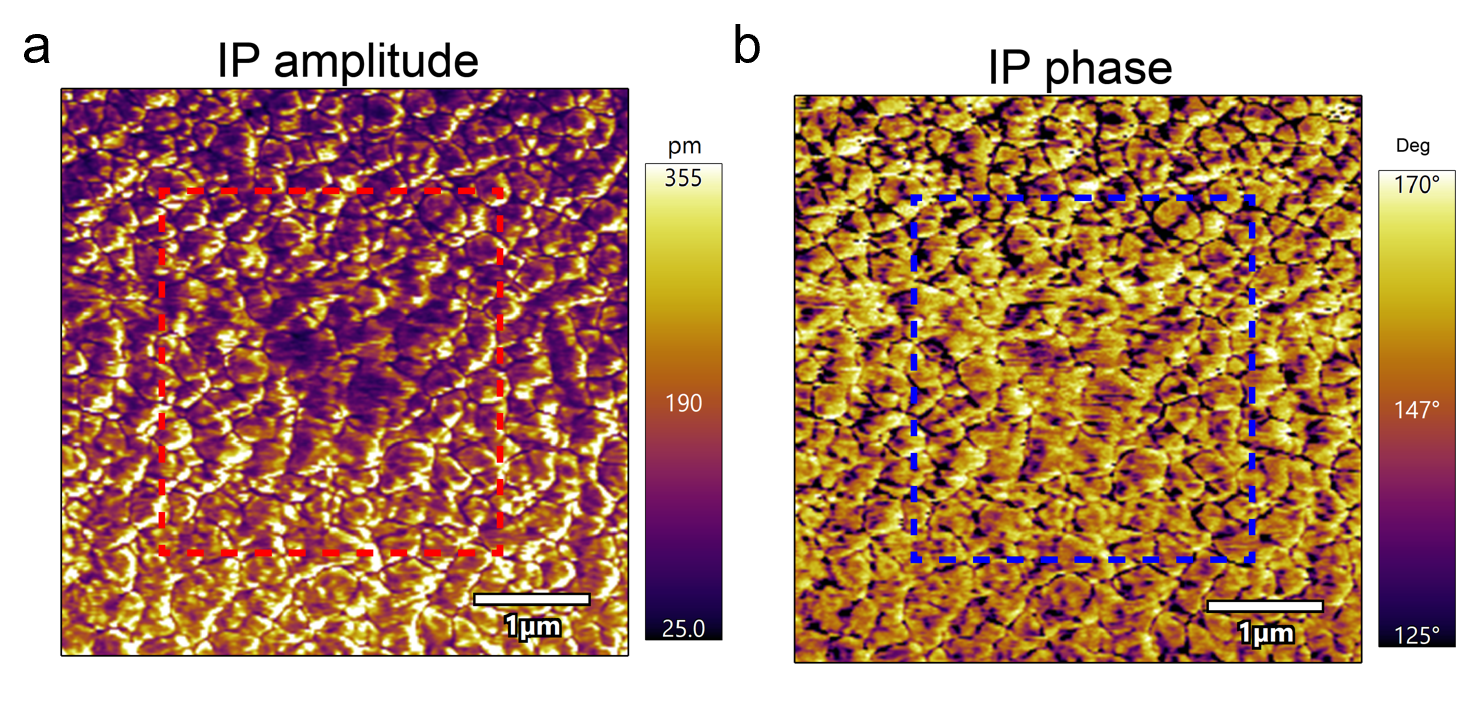


**Figure S12| In-plane piezoresponse force microscopy (PFM) characterization of the control CsFAMA sample.**

**a.** Amplitude image and **b.** phase image.

After repeated scanning under a DC bias of ±8 V, no significant differences in amplitude or phase contrast were observed between the electrically poled and unpoled regions, indicating the absence of a stable ferroelectric response in the control sample under the applied conditions.


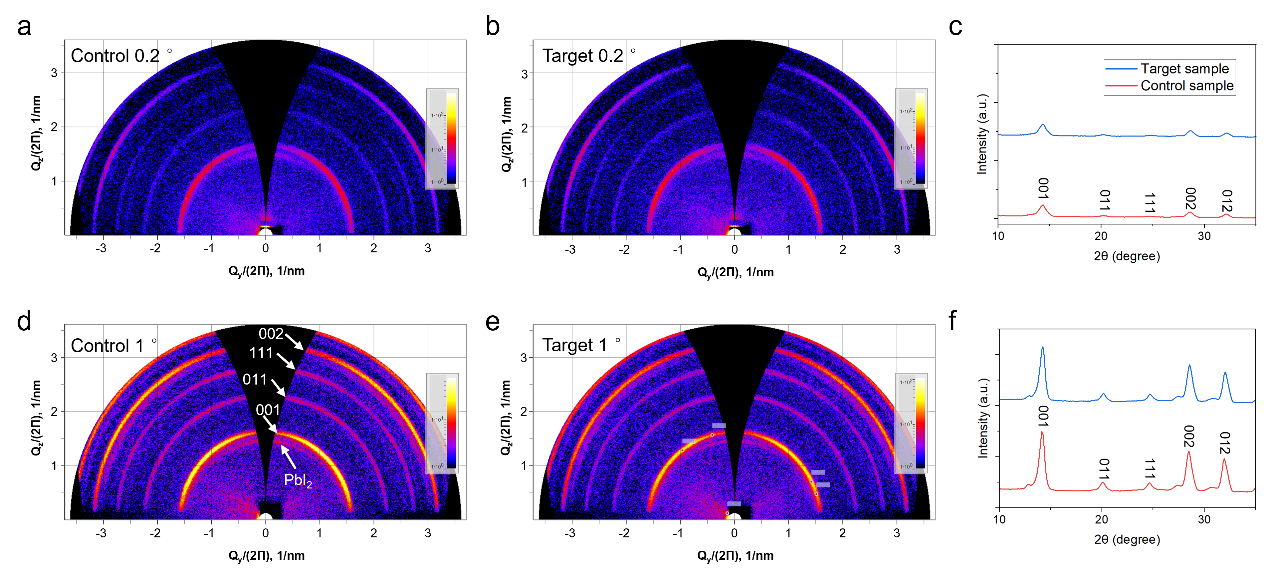


**Figure S13| Structural integrity analysis via Grazing-Incidence Wide-Angle X-ray Scattering (GIWAXS).**

**a, b**. 2D GIWAXS patterns of the control (a) and target (b) films collected at a grazing incidence angle of 0.2° (surface-sensitive mode).

**d, e**. 2D GIWAXS patterns of the control (d) and target (e) films collected at an incidence angle of 1.0° (bulk-sensitive mode).

**c, f**. Corresponding azimuthally integrated 1D X-ray diffraction intensity profiles derived from the 0.2° (c) and 1.0° (f) patterns.

The diffraction peaks exhibit identical positions and relative intensities for both samples. The absence of detectable peak shifts or new phases confirms that the trace incorporation of EFC preserves the bulk lattice parameters and high crystallinity of the perovskite host, implying that the polymer selectively modulates the grain boundaries or interfaces rather than intercalating into the lattice.


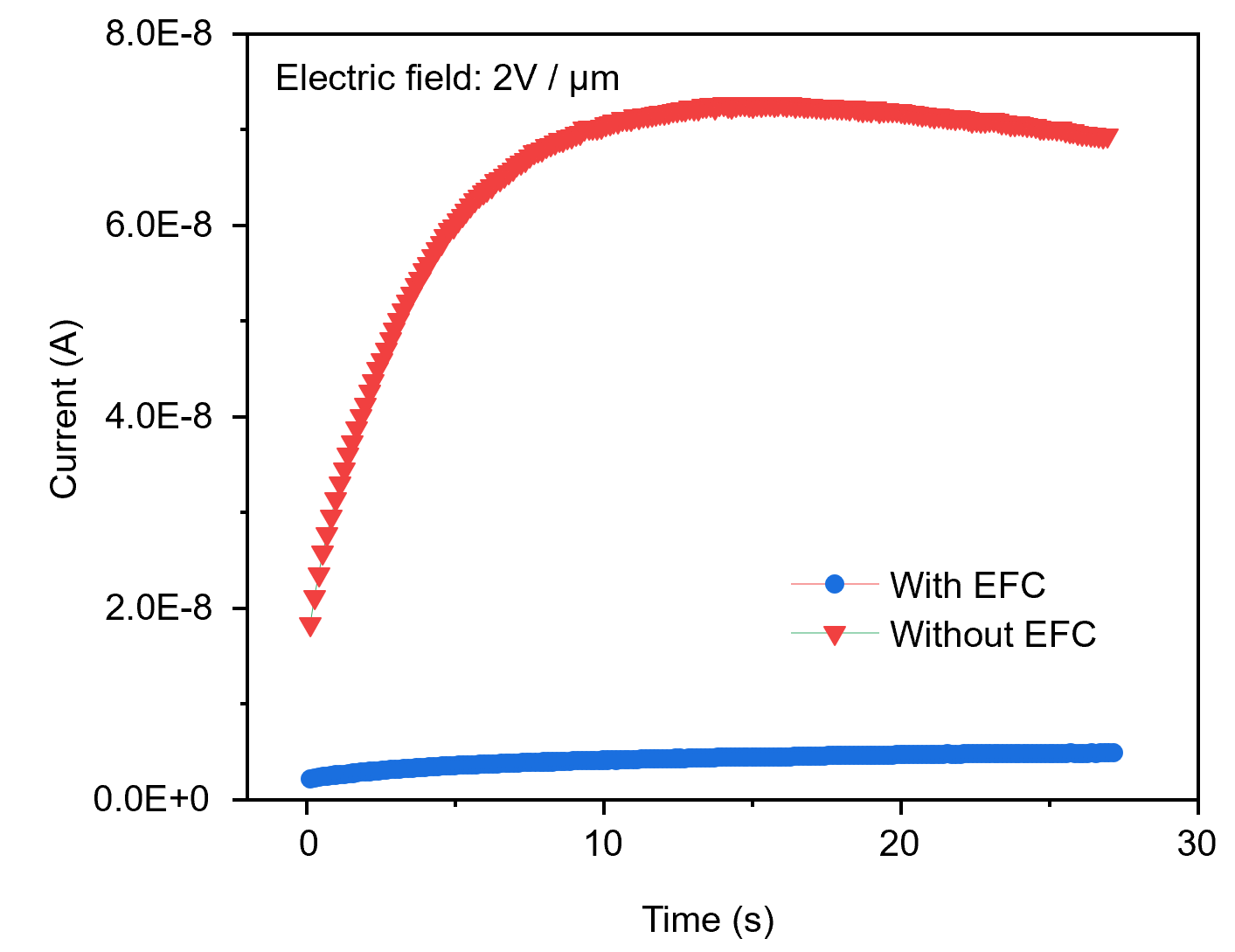


**Figure S14| The dark current of both the control and target samples.**

Under identical electric field conditions, the dark current of both the control and target samples was measured. When tested under an electric field of 2 V/μm, the control sample exhibited a more rapid current increase, and a higher current magnitude compared to the target sample.

This difference is attributed to the incorporation of EFC into the perovskite film, which leads to a reduction in conductivity. As an electrically insulating polymer, EFC does not contribute free carriers and may even form high-resistance regions within the bulk or near grain boundaries, thereby increasing interface impedance and introducing carrier transport barriers. These findings indirectly confirm that the EFC has been successfully incorporated into the perovskite film and is actively influencing its electrical properties.


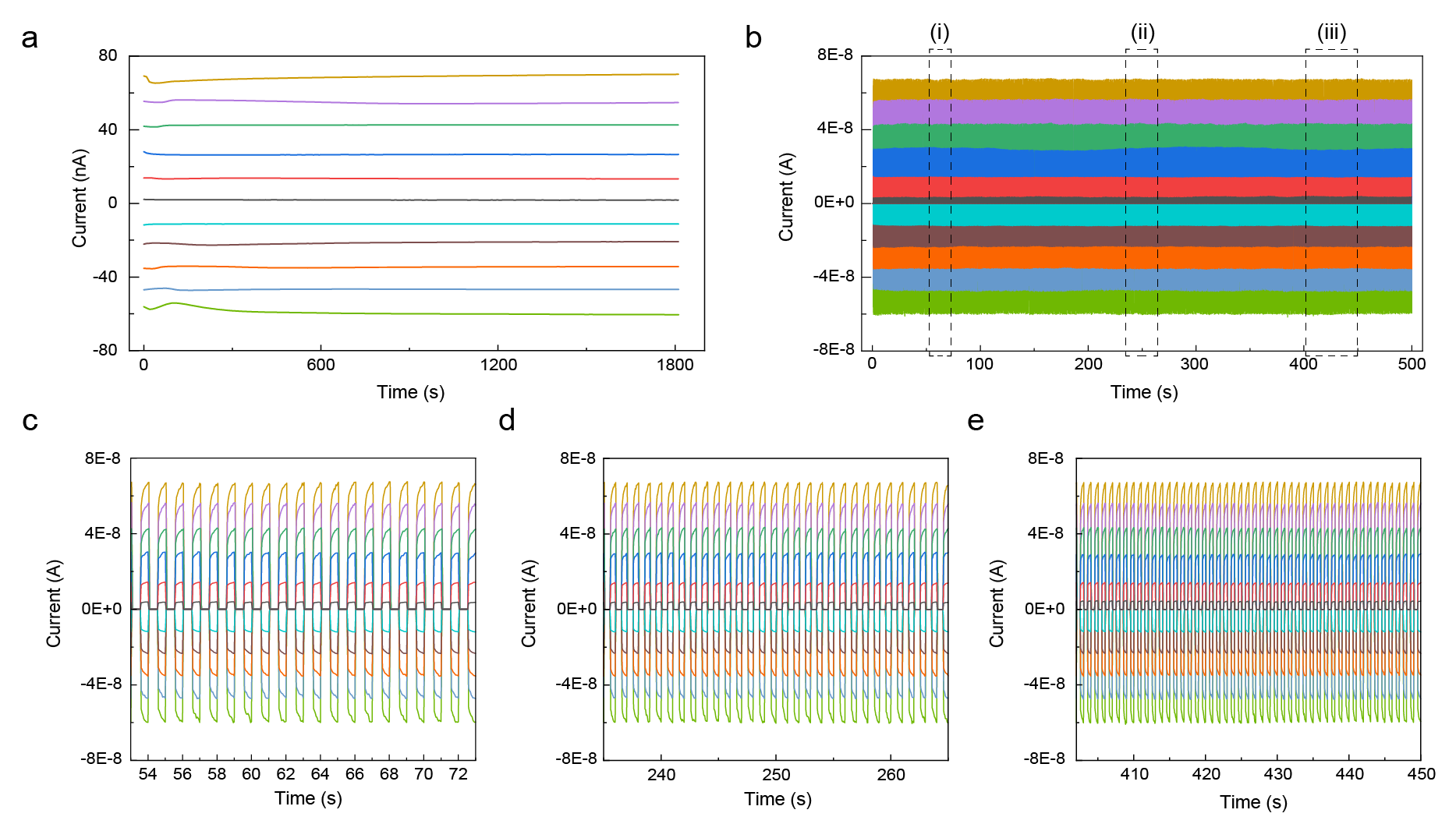


**Figure S15| Retention and stability of programmed photoresponsivity states.**

**a**. Photocurrent (I_sc_) measured at 0 V under continuous illumination (50 mW cm^-2^) for 1800 s after programming with different electric fields. All programmed states remain clearly distinguishable and stable, indicating good retention under continuous optical operation.

**b**. Cyclic light pulses responses of the corresponding programmed states under 0.5 s on / 0.5 s off illumination for 500 cycles. No obvious state drift is observed during repeated optical readout.

**c-e**. Enlarged views of the dashed regions in b at (i) 53-73 s, (ii) 235-265 s, and (iii) 403-450 s, respectively, further confirming the highly stable photocurrent amplitude and waveform of each programmed state.


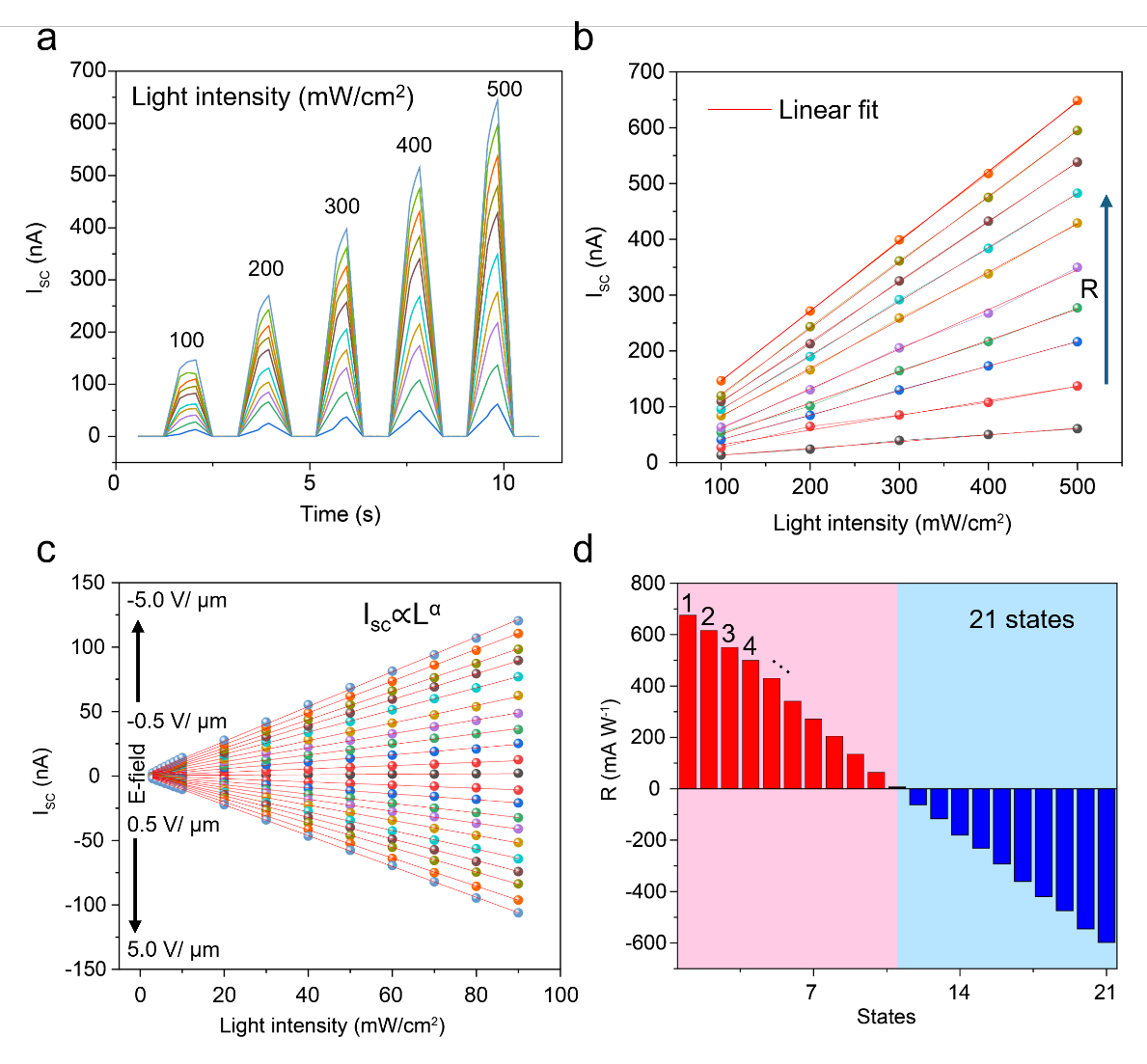


**Figure S16| Analysis of photoresponsivity under high-intensity illumination and photoresponsivity states.**

**a.** After electric field programming at various levels, the I_sc_ of the device was measured under light pulses with increasing intensity ranging from 100 to 500 mW/cm².

**b.** The device maintains a linear I_sc_ response even at high light intensities, indicating excellent linearity between I_sc_ and light intensities (ranging from 3 to 100 mW/cm²).

**c.** I_sc_ of the target device under programmed electric fields from ±0.5 V/μm to ±5 V/μm as a function of incident light intensity (L), corresponding to distinct photoresponsivity states. The data were fitted using a power-law relationship $I_{sc}\propto L^{\alpha}$, where $\alpha$ represents the light-response exponent. For negatively programmed states, the fitting was performed on the magnitude of the photocurrent (|$I_{sc}$|). The extracted $\alpha$ values as below, confirming sublinear photoresponse behavior in the photovoltaic (zero-bias) mode.

| Programmed electric fields (V/μm) | -0.5 | -1.0 | -1.5 | -2.0 | -2.5 | -3.0 | -3.5 | -4.0 | -4.5 | -5.0 |
| --- | --- | --- | --- | --- | --- | --- | --- | --- | --- | --- |
| *α* | 0.95 | 0.93 | 0.93 | 0.98 | 1.01 | 0.96 | 1.04 | 0.97 | 0.97 | 1.03 |
| Programmed electric fields (V/μm) | 0.5 | 1.0 | 1.5 | 2.0 | 2.5 | 3.0 | 3.5 | 4.0 | 4.5 | 5.0 |
| *α* | 0.93 | 0.97 | 0.92 | 0.94 | 1.03 | 0.95 | 0.93 | 0.93 | 0.96 | 0.98 |

**d.** A total of 21 resolvable photoresponsivity levels were obtained, corresponding to R value: 675.5 mA W^-1^, 616.5 mA W^-1^, 550 mA W^-1^, 500 mA W^-1^, 429.5 mA W^-1^, 341 mA W^-1^, 272 mA W^-1^, 204.5 mA W^-1^, 135 mA W^-1^, 64 mA W^-1^, 7.5 mA W^-1^, -61.9 mA W^-1^, -116.35 mA W^-1^, -181.25 mA W^-1^, -231.15 mA W^-1^, -293.35 mA W^-1^, -360.75 mA W^-1^, -419.65 mA W^-1^, -474.45 mA W^-1^, -545.35 mA W^-1^, -597.9 mA W^-1^.


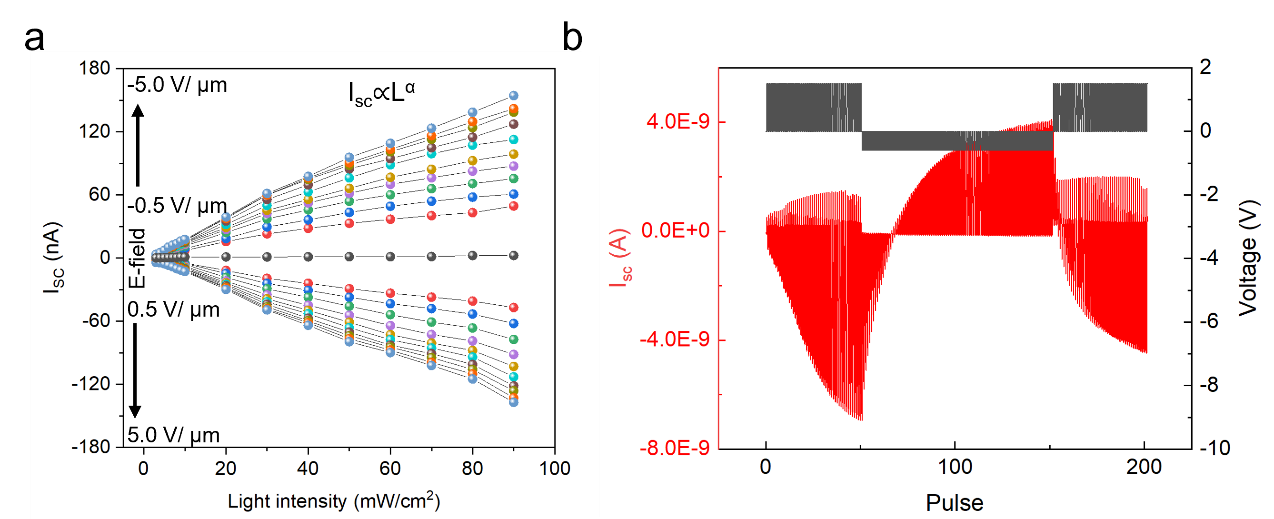


**Figure S17| Analysis of photoresponsivity controllability in the control sample.**

1. I_sc_ distribution of the control sample after each electric field programming step (electric fields from ±0.5 V/μm to ±5 V/μm). The data were fitted using a power-law relationship $I_{sc}\propto L^{\alpha}$,with the fitted $\alpha$ values as below:

| Programmed electric fields (V/μm) | -0.5 | -1.0 | -1.5 | -2.0 | -2.5 | -3.0 | -3.5 | -4.0 | -4.5 | -5.0 |
| --- | --- | --- | --- | --- | --- | --- | --- | --- | --- | --- |
| *α* | 0.83 | 0.85 | 0.83 | 0.88 | 0.84 | 0.86 | 0.81 | 0.82 | 0.86 | 0.85 |
| Programmed electric fields (V/μm) | 0.5 | 1.0 | 1.5 | 2.0 | 2.5 | 3.0 | 3.5 | 4.0 | 4.5 | 5.0 |
| *α* | 0.83 | 0.88 | 0.86 | 0.86 | 0.82 | 0.81 | 0.83 | 0.81 | 0.84 | 0.84 |

**b.** I_sc_ response of the control sample under continuous electrical pulse stimulation. illumination intensity:50 mW cm^-2^, light wavelength: 623 nm.

Although the control sample exhibits both positive and negative photoresponsivity, its intrinsic rapid ion migration leads to a decay in photocurrent during testing. As a result, a clear deviation is observed between the simulated response curve and the measured data. Moreover, the use of continuous pulses fails to produce linearly tunable and symmetric current responses. These behaviors highlight the instability and poor controllability of the control sample, rendering it unsuitable for further application in programmable photoresponsive.


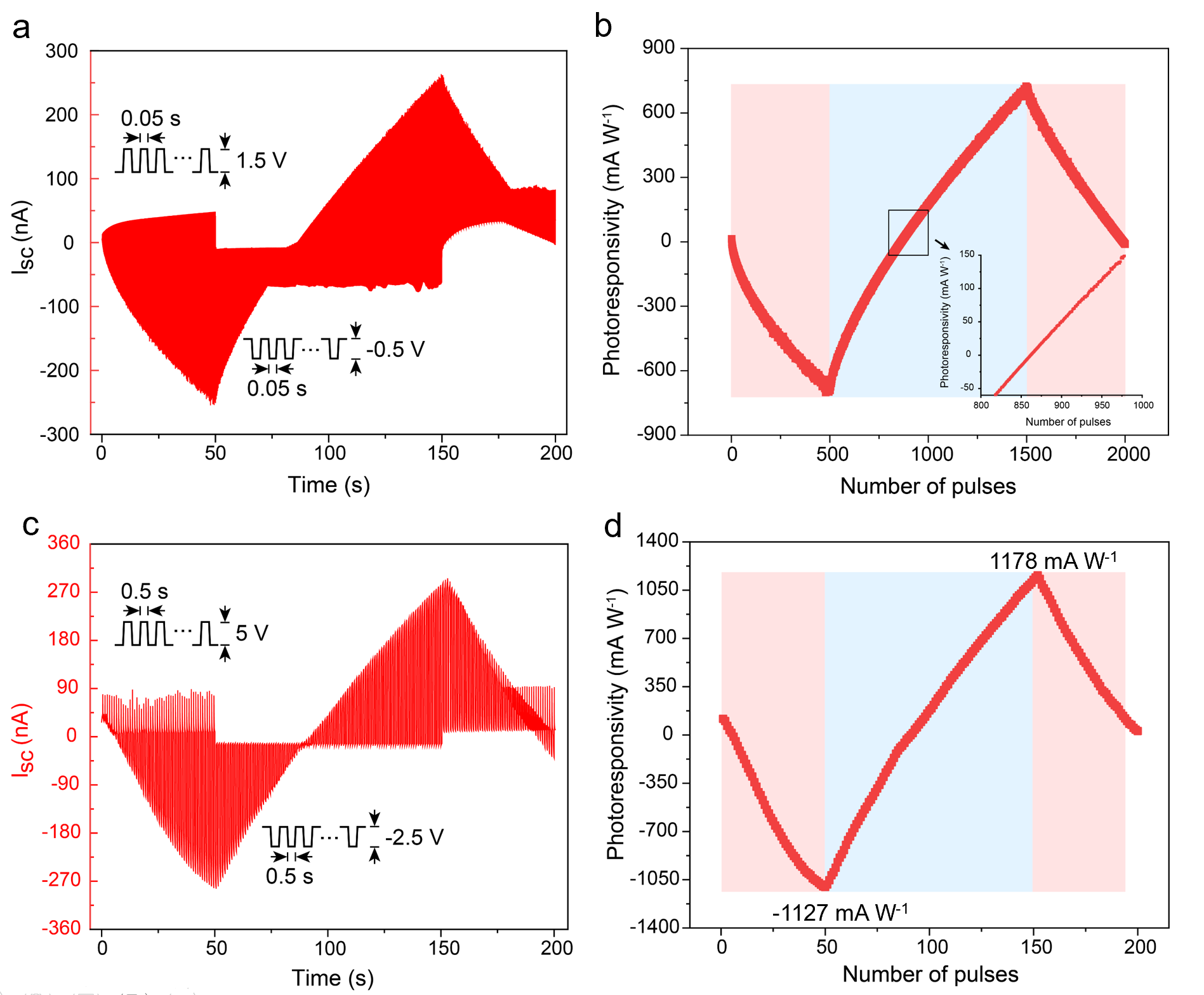


**Figure S****18| Analysis of photoresponsivity controllability in the target sample.**

**a.** The I_sc_ of the target sample exhibits a linear and stable increase under continuous pulse programming, demonstrating excellent tunability. illumination intensity: 50 mW cm^-2^, light wavelength: 623 nm. positive pulses with 1.5 V amplitude and 0.5 s width, negative pulses with -0.5 V amplitude and 0.05 s.

**b.** The extracted I_sc_ values are converted into photoresponsivity (R) values using Equation 1:

$$R=\frac{I_{SC}}{P}$$

where I_sc_ is the short-circuit photocurrent and $P$ is the incident light power.

It is important to note that $P$ refers to light power, not light intensity. The total light power was calibrated using a Thorlabs PM100D optical power meter. The beam diameter at the sample plane was measured, and the corresponding light density was calculated from the total power and beam area. Then, the effective optical power incident on the device was calculated according to the active area. Therefore, $P$ is determined according to Equation 2:

$$P=light intensity\times S_{active area of the device}$$

In this case, the light intensity is 0.5 mW/mm^2^(50 mW cm^-2^), and the active area is 10 μm × 50 μm (The illuminated area corresponds to the lateral channel region between the electrodes (10 μm × 50 μm), which is fully covered by the incident light spot). Inset image shows 200 states.

**c.** The I_sc_ of the target sample exhibits a linear and stable increase under continuous pulse programming, demonstrating excellent tunability. illumination intensity:50 mW cm^-2^, light wavelength: 623 nm. positive pulses with 5 V amplitude and 0.5 s width, negative pulses with -1.5 V amplitude and 0.5 s.

**d.** The extracted I_sc_ values of figureS18c.


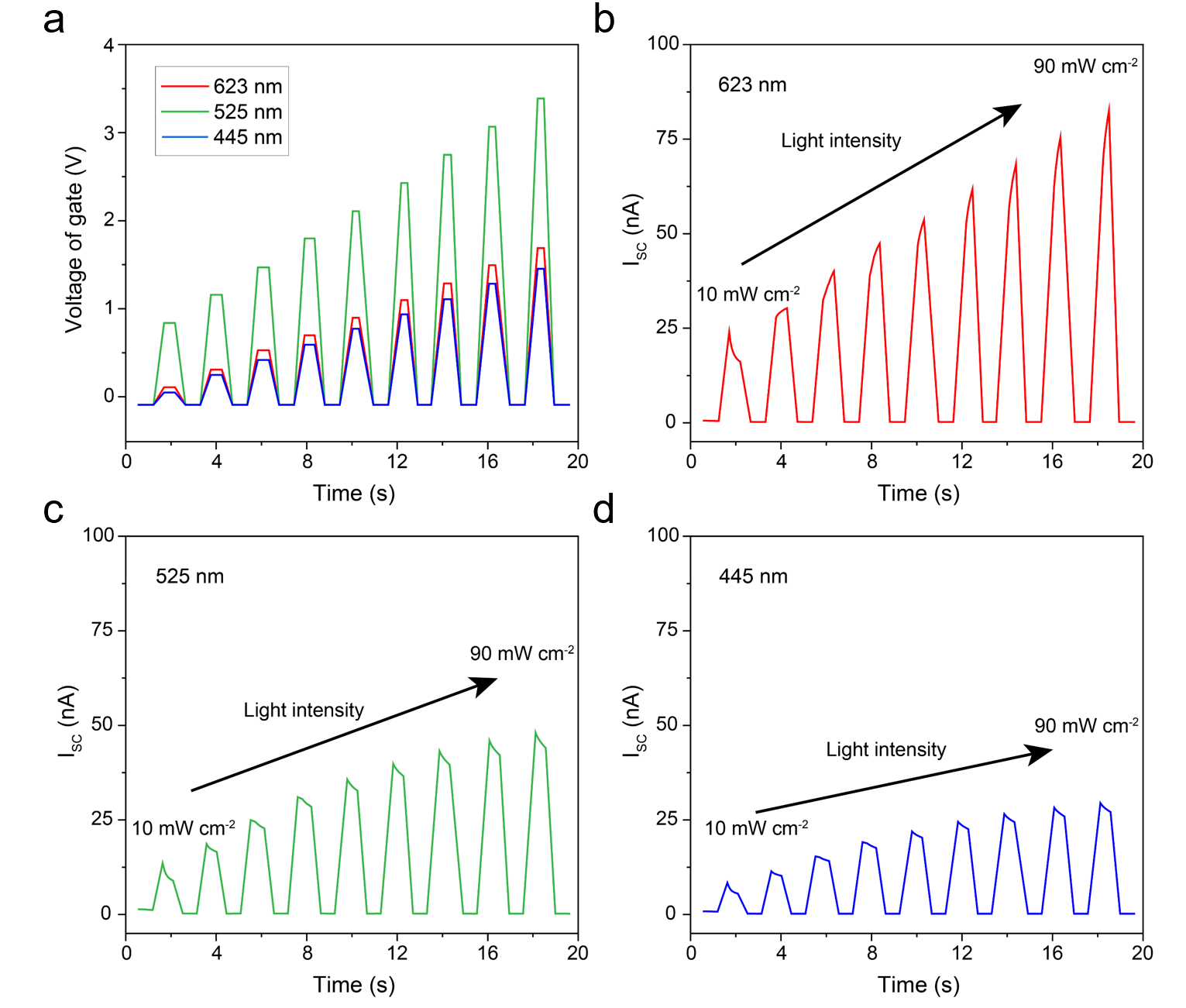


**Figure S19| Multi-wavelength photoresponse characteristics of the target sample.**

**a.** Different programming voltage pulses were applied to the light-controller, while maintaining the same light intensity for three distinct wavelengths.

**b–d.** I_sc_ responses of the device under illumination at 623 nm, 525 nm, and 445 nm, respectively. The results demonstrate that the device exhibits a robust and tunable photoresponse across multiple wavelengths, highlighting its potential for broad-spectrum optoelectronic applications.

**Table S2. Benchmark the performance of the perovskite-EFC photovoltaic devices against other recent state-of-the-art works.**

| **Materials** | **Terminal** | **Max R (mA/W)** | **Response time** | **States** | **Retention** | **Linear of I_sc_-Light intensity** |
| --- | --- | --- | --- | --- | --- | --- |
| 3R-WS_2_ ^4^ | 2 | 915 | 12.8 μs | 30 | 1000s | 1 - 200 mW/cm^2^ |
| Epitaxial PZT ^8^ | 2 | 0.6 | 30 μs | 21 | 50 days | 85 mW/cm^2^ |
| MoS_2-x_ ^2^ | 2 | 369 | \ | 11 | 1000s | 16 - 2600 mW/cm^2^ |
| MoTe_2_/P(VDF-TrFE) ^3^ | 4 | 830 | 0.6 μs | 51 | 1000s | 150 - 717 mW/cm^2^ |
| PdSe_2_/MoTe_2_ ^11^ | 3 | 350 | 0.4 μs | 6 | volatile | 381 mW/cm^2^ |
| Al2O_3_/HfO_2_/Al2O_3_/ black phosphorous ^10^ | 3 | 60 | \ | 8 | 2000s | \ |
| WSe_2_ ^7^ | 4 | 60 | ＜40 ns | \ | volatile | \ |
| WSe_2_/P(VDF-TrFE) ^14^ | 4 | \ | \ | 82 | 150s | 2 - 8 mW/cm^2^ |
| SWNT@GDY ^6^ | 3 | 30 | 5 ms | 32 | 10000s | 10 - 1200 mW/cm^2^ |
| WSe_2_/Al_2_O_3_/HfO_2_/Al_2_O_3_ ^5^ | 4 | 50 | 5 μs | 64 | 1000s | 25 - 50 mW/cm^2^ |
| WS_2_ nanotubes ^1^ | 2 | \ | 64 μs | \ | 10 days | \ |
| WSe_2_/h-BN/Al2O_3_ ^15^ | 3 | \ | 8 ms | \ | \ | \ |
| MoS_2−x_O_x_ ^12^ | 2 | 0.01 | 1.38 ms | 7 | 3000s | \ |
| NbS_2_/MoS_2_ ^16^ | 3 | 1100 | 304 ms | 15 | volatile | 240 mW/cm^2^ |
| PEDOT:PSS/FA_0.8_Cs_0.2_Pb_0.5_Sn_0.5_I_3_/C_60_/BCP ^17^ | 3 | 1110 | 5.4 ms | 32 | \ | 0.01 – 0.2 mW/cm^2^ |
| **This work** | **2** | **1120** | **730 μs** | **1000** | **1800s** | **3 - 500 mW/cm^2^** |


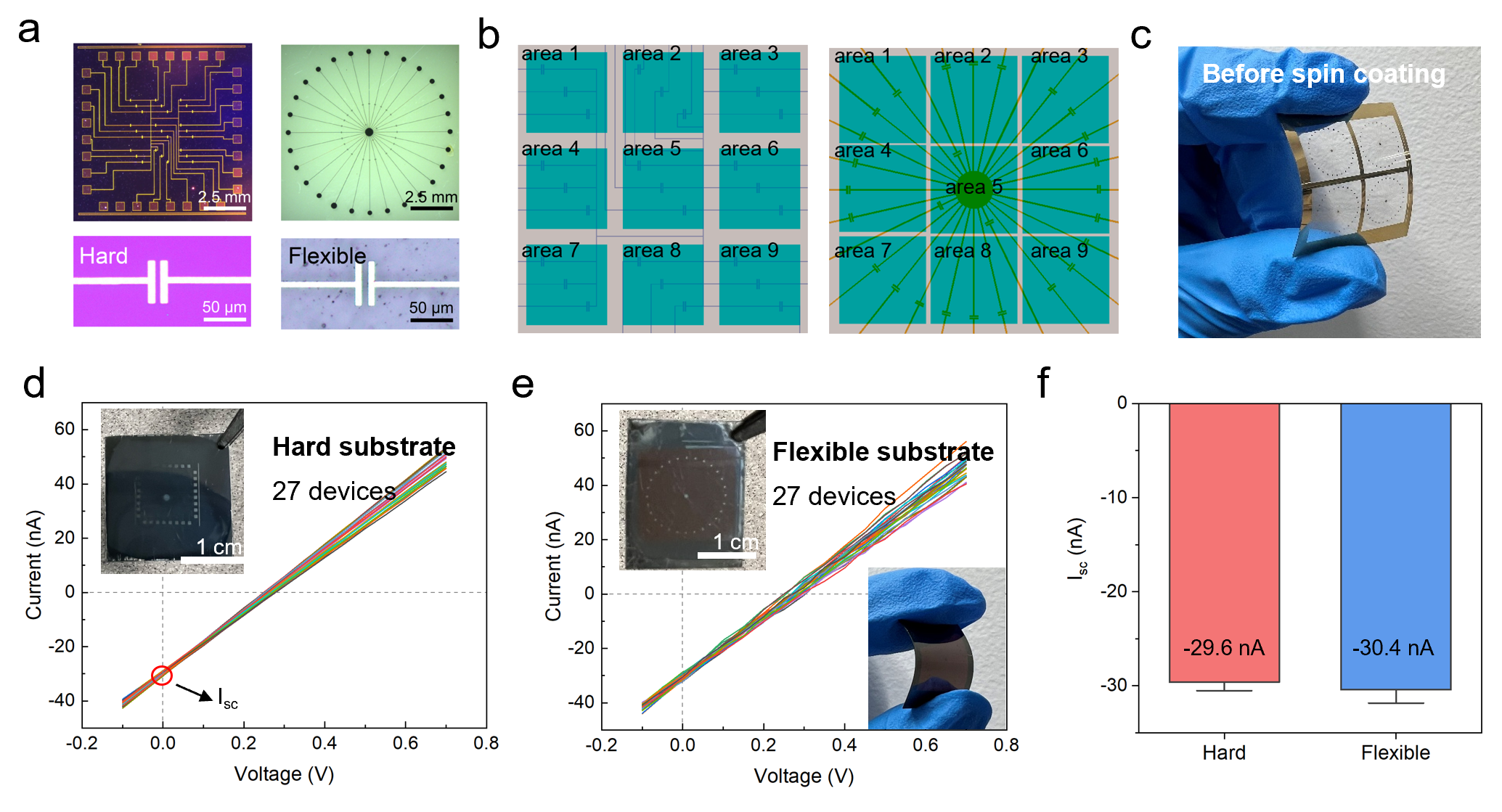


**Figure S20| Demonstrating substrate compatibility and device uniformity.**

**a.** The optical image of the array, which contains 27 individual devices, zoom in area shows the optical image of single lateral device with an electrode length of 50 um and a gap width of 5 μm.

**b.** A total of 27 individual lateral devices are distributed across 9 functional areas. The left design utilizes a square-shaped hard substrate, which is suitable for integration with peripheral circuitry. The right design adopts a circular flexible substrate, with devices oriented perpendicular to the diameter to accommodate bending and mechanical stress during practical use.

The 9 regions of the array can function as a 3 × 3 convolution kernel, with each region containing 3 individual devices. Therefore, the array is capable of simultaneously operating as three independent 3 × 3 convolution kernels, enabling parallel processing of multiple input features.

**c.** Optical image of the flexible electrode device array before perovskite deposition.

**d.** I_sc_ measurements of devices on hard and **e.** flexible substrates under a programming electric field of 3 V/μm. Inset images are devices after spin coating perovskite.

**f.** Statistical results of the I_sc_ were obtained from 27 devices in arrays fabricated on different substrates.

The comparison demonstrates the substrate-independent performance uniformity, confirming the compatibility and reliability of the device architecture across both rigid and flexible platforms.


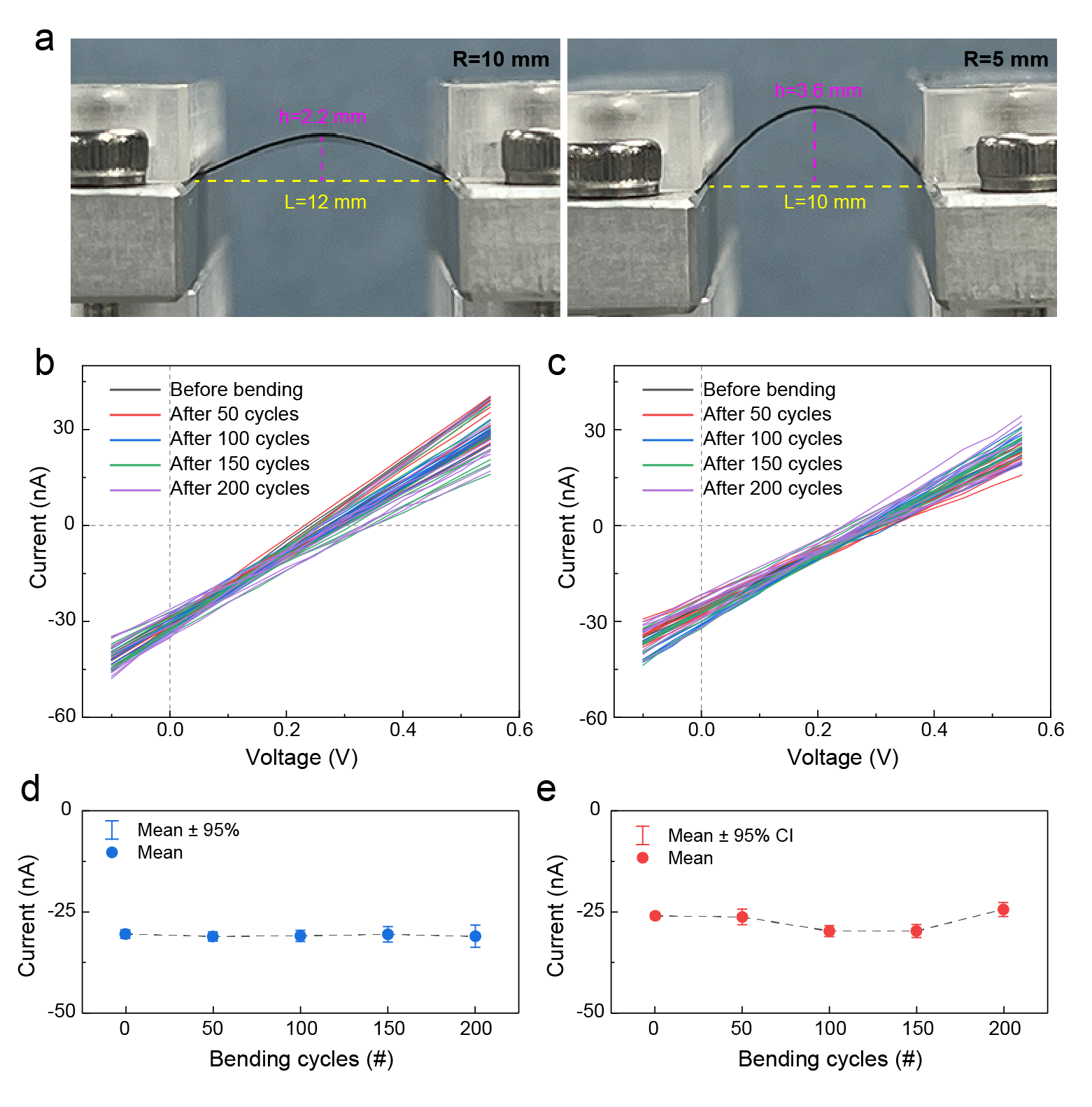


**Figure S21| Evaluation of mechanical bending effects on the flexible device performance.**

**a**. Side-view optical images of the bent flexible device with estimated bending radii of 10 mm and 5 mm, respectively, calculated from the chord length (L) and bending height (h). **b, c**. *I-V* curves of representative devices from the flexible array, measured after electrical programming at the flat state (0 cycle) and after repeated bending every 50 cycles up to 200 cycles. **d, e**. Corresponding I_sc_ distributions extracted from the programmed device under the two bending conditions. The relatively stable I_sc_ values indicate good mechanical robustness of the flexible device under repeated deformation.


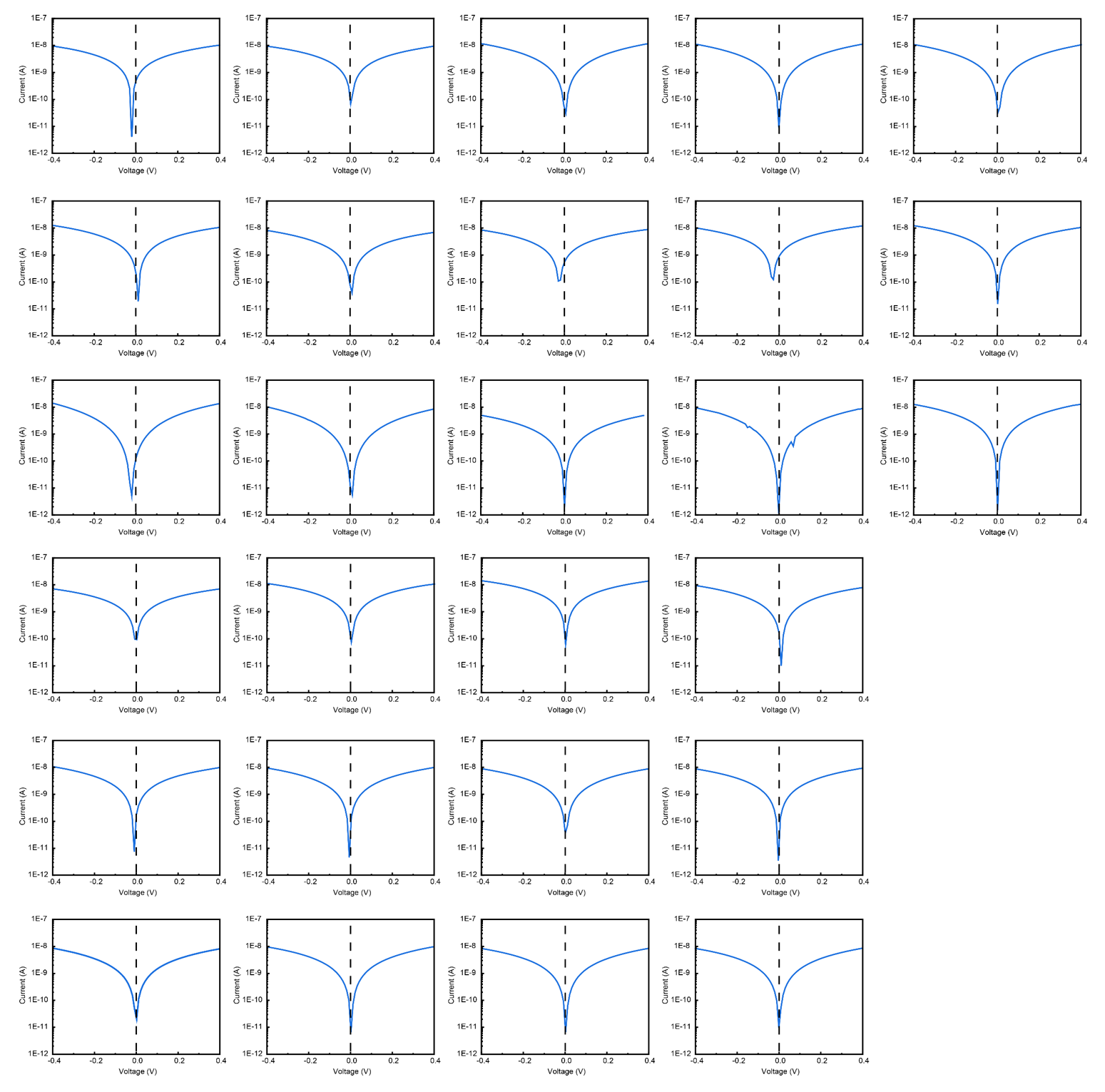


**Figure S22| Evaluation of the initial state of the array devices.**

To ensure the proper functioning of the array, it is essential that all individual devices exhibit a stable and consistent initial state. Before programming, all 27 devices in the array demonstrated normal electrical behavior, with no significant shift in V_oc_, and I_sc_ levels below the nanoampere range. These results confirm the uniformity of the array design, validating its suitability for the subsequent photoresponsivity programming process.


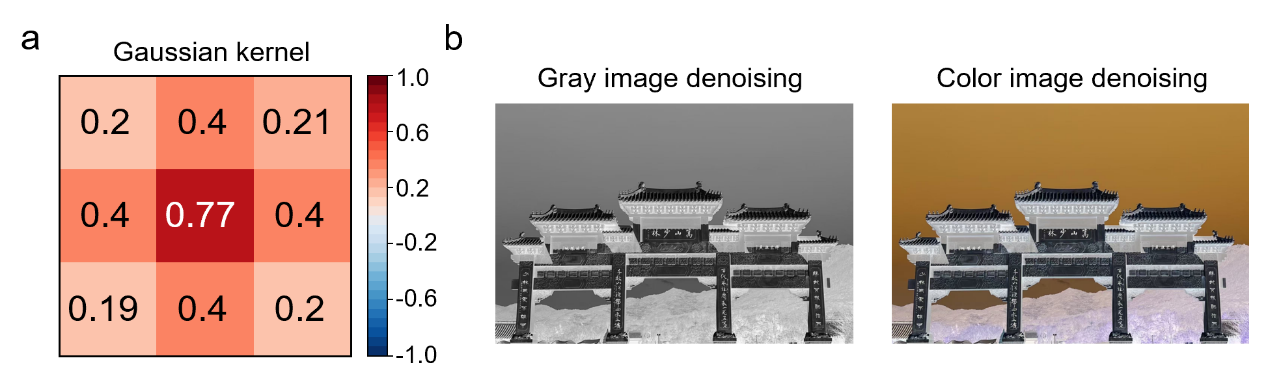


**Figure S23| Image denoising using a Gaussian convolution kernel.**

**a.** The photoresponsivity values programmed into the array are used to construct a Gaussian convolution kernel.

**b.** Results of the denoising convolution operation applied to both the original image and its grayscale version.

The programmable photoresponsivity range of the device enables the implementation of various convolution kernels, allowing flexible adjustment of weights to perform different image processing functions. As each kernel produces distinct effects, they can be tailored for specific application scenarios, such as denoising, edge enhancement, or feature extraction.


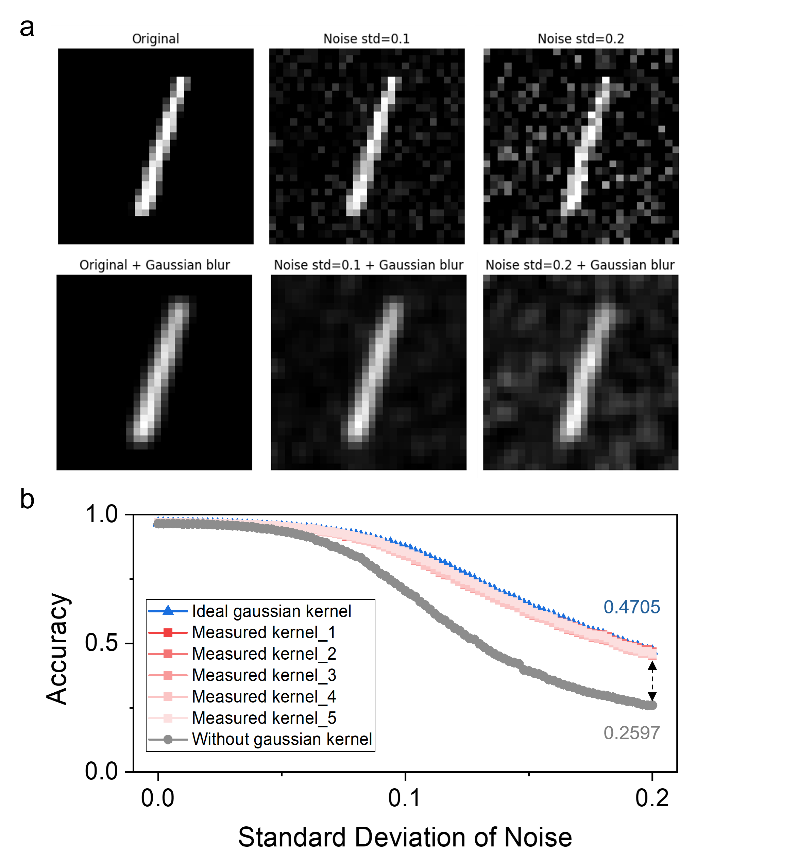


**Figure S24| Evaluate the denoising effect of the Gaussian kernel-based CNN system.**

**a.** The original image of the MNIST digit 1 and the effects of adding different levels of noise (noise standard deviation = 0.1, 0.2, top images). The result of processing the noisy image with Gaussian kernel, showing the denoising ability (bottom images).

**b.** Accuracy changes with the introduction of noise, using Gaussian kernels under different conditions (ideal, measured) and compared with no Gaussian kernel network.

The Gaussian kernel obtained from the experiment has the same effect as the ideal Gaussian kernel. When the noise standard deviation is 0.2, the accuracy drops to about 47%. The neural network without CNN has even weaker noise resistance, with the accuracy dropping to only 25.97%.


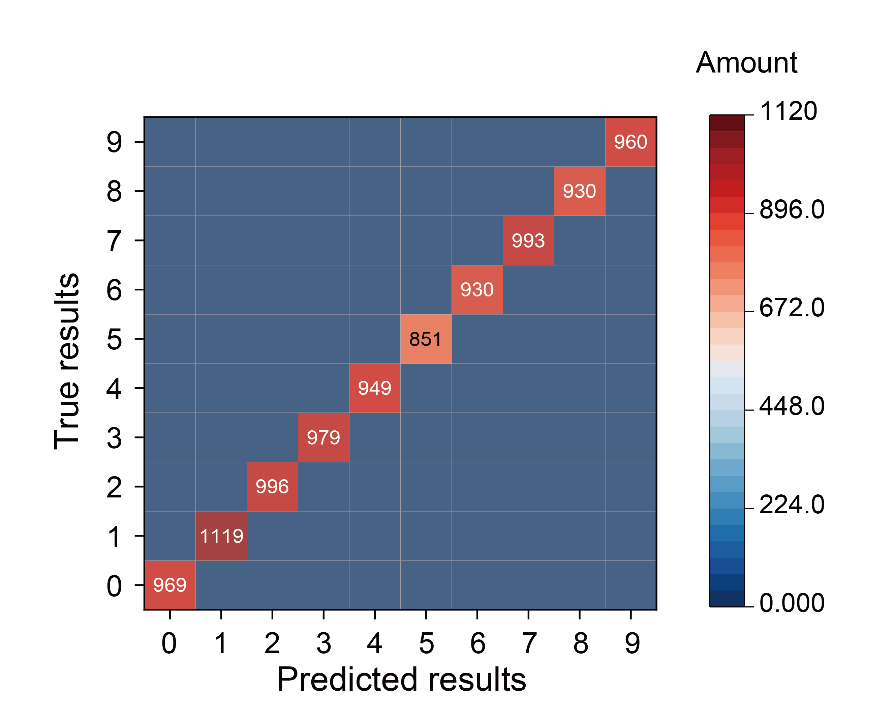


**Figure S25| Confusion matrix of the MNIST classification task using software simulation.**

In the software-based convolutional neural network simulation for MNIST classification, the convolutional kernel is an ideal Gaussian kernel and the weights of fully connected layer are dynamically updated during training to optimize accuracy.

**
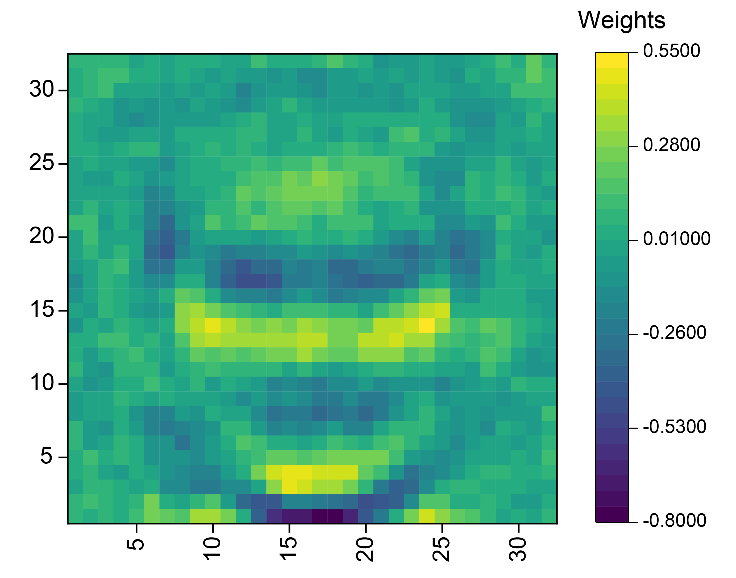
**

**Figure S26| The 32×32 confusion matrix of a single-stage classifier for face detection.**

This kernel serves as a linear spatial feature extractor that responds strongly to structural patterns typical of human faces, such as eyes, nose bridge, and mouth, by assigning higher weights to pixel groupings that resemble these features. The output of the convolution (a scalar value) represents the confidence score for the presence of a face. This value is then compared against a threshold to make a binary decision: “face” or “non-face”.

Importantly, this kernel was optimized using the FDDB dataset to achieve high recall (>95%) under relaxed thresholds, ensuring minimal omission of true faces at the cost of some false positives. This bias toward sensitivity makes it ideal for early-stage filtering in a hierarchical visual system, where downstream processing (e.g., DNNs) can further refine the decision.


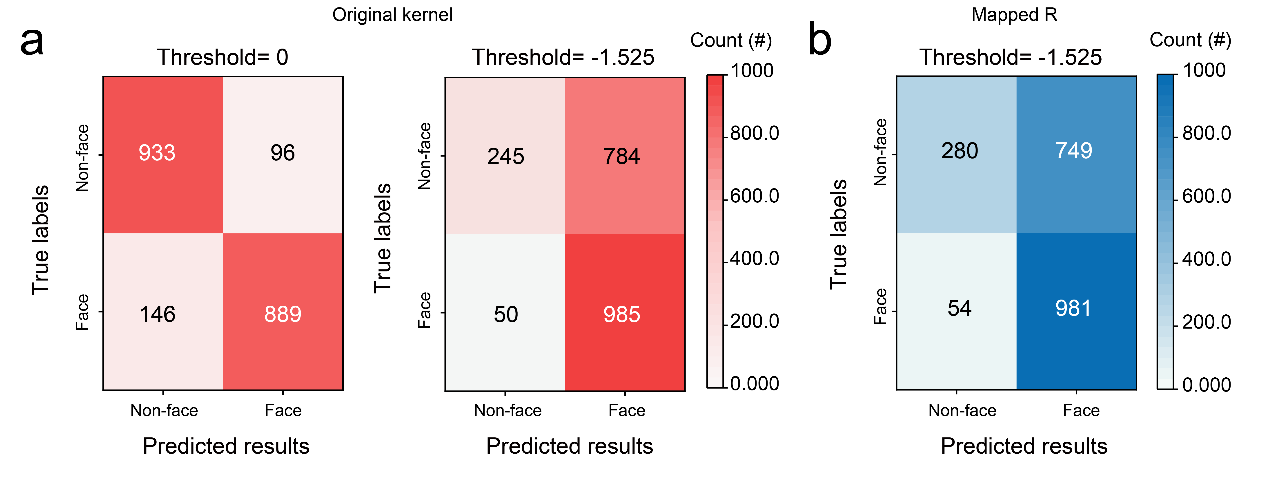


**Figure S27| Confusion matrices using the original and mapped kernel for 2064 patches with different thresholds.**

**a.** At threshold = 0, the system achieves balanced performance (Accuracy: 88.28%; Precision: 0.9025; Recall: 0.8589; F1-score: 0.8802). Lowering the threshold to -1.525 improves recall (0.9517) but reduces precision.

**b.** Confusion matrices using the measured photoresponsivity-based kernel. At threshold = -1.525, the system achieves balanced performance (Accuracy: 61.09%; Precision: 0.5671; Recall: 0.9478; F1-score: 0.7096).


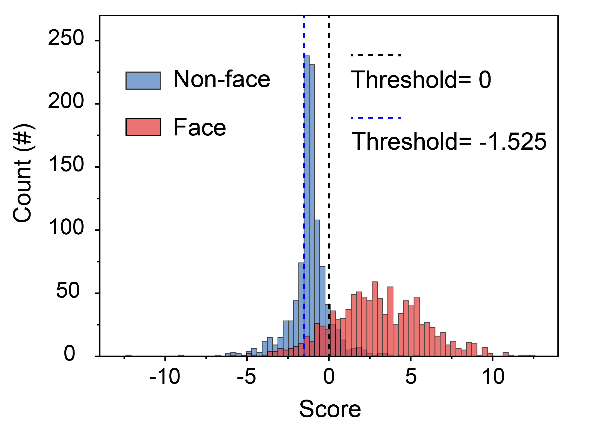


**Figure S28| The distribution of scores using the original kernel.**

To evaluate the functional behavior of the trained 32×32 convolutional kernel, we analyzed the distribution of its convolutional output scores over a dataset of 32×32 grayscale image patches, which includes both face and non-face samples derived from the FDDB dataset.

The resulting score distribution exhibits the following characteristics:

Face patches tend to produce higher scores, as their structural patterns (e.g., symmetric contours, eye/nose regions) align well with the kernel’s learned weights. Non-face patches generally yield lower scores, as they lack the spatial correlations emphasized by the kernel.

The relatively clear margin between face and non-face score distributions confirms that the kernel performs effective early-stage discrimination and is well-suited for hardware-level implementation in neuromorphic vision systems.

**Supplementary Note 1| Evaluation Metrics for Binary Classification.**

In the context of distinguishing face from non-face image patches, performance evaluation is typically based on four fundamental outcomes:

**TP:** a face, correctly identified as a face (True Positive)

**TN:** a non-face, correctly identified as non-face. (True Negative)

**FP:** a non-face, incorrectly classified as a face. (False Positive)

**FN:** a face, incorrectly classified as a non-face. (False Negative)

From these quantities, several widely adopted performance metrics can be derived:

1. **Accuracy** indicates the overall correctness of the model. It reflects the proportion of correctly classified samples among all predictions. However, in imbalanced datasets (e.g., with far more non-faces than faces), accuracy alone may not fully capture the model’s effectiveness.

$$Accuracy=\frac{TP+TN}{TP+TN+FP+FN}$$

2. **Precision** (Positive Predictive Value) quantifies the proportion of predicted faces that are correct. High precision means fewer false positives, which is important in applications where false alarms are costly.

$$Precision=\frac{TP}{TP+FP}$$

3. **Recall** (Sensitivity or True Positive Rate) measures the proportion of actual faces that are successfully detected. A high recall is critical when missing a face (e.g., in a security system) is more problematic than triggering a false alarm.

$$Recall=\frac{TP}{TP+FN}$$

4. The **F1-score** is the harmonic means of precision and recall. It provides a single metric that balances the trade-off between precision and recall. It is particularly useful when both false positives and false negatives are important to consider.

$$F1=2\times\frac{Precision\times Recall}{Precision+Recall}$$

5. **Specificity** (True Negative Rate) measures the proportion of actual non-face samples that are correctly identified as non-faces. In other words, it reflects the model's ability to avoid false alarms.

$$Specificity=\frac{TN}{TN+FP}$$

In our work, these metrics help quantitatively assess how well the convolutional kernel, whether original or device-mapped, performs in early-stage face detection. And for an energy-efficient vision system, tuning the decision threshold allows for a flexible balance between recall and specificity, depending on whether the system prefers to be sensitive (detect more faces) or precise (avoid false positives).

**Supplementary Note 2 | ROC Curve and AUC.**

The **Receiver Operating Characteristic (ROC)** Curve is a graphical plot that illustrates the performance of a binary classifier system as its discrimination threshold is varied. It is generated by plotting the True Positive Rate (TPR) against the False Positive Rate (FPR) at various threshold settings.

**True Positive Rate (TPR)** (also known as Recall or Sensitivity):

$$TPR=\frac{TP}{TP+FN}$$

**False Positive Rate (FPR)**

$$FPR=\frac{FP}{FP+TN}$$

Each point on the ROC curve corresponds to a particular threshold used for classification. A curve closer to the top-left corner indicates a better-performing model, with high sensitivity and a low false positive rate.

The **Area Under the ROC Curve (AUC)** represents the area under the ROC curve, which serves as a scalar metric summarizing the overall performance of a classifier across all thresholds. Its value ranges from 0.5 (random guess) to 1.0 (perfect classification). A higher AUC reflects a better balance between sensitivity and specificity. In our study, AUC was used to quantitatively assess the degradation or stability of classification performance under different physical implementations of the kernel (e.g., original weights vs. mapped vs. noisy), as shown in Figure 6i.

**Generation Method of Randomized Noisy Kernels**

To emulate the real-world variability in device programming, especially the write noise across multiple devices, we introduced a randomized kernel sampling method for performance validation under noise. The procedure is as follows:

Ten groups of device-programmed photoresponsivity values were experimentally collected, corresponding to the same target kernel values.


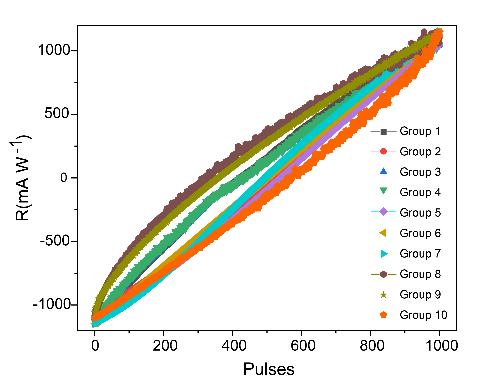


For each element in the 32×32 kernel (i.e., total 1024 weights), a random integer between 1 and 10 was generated. Based on this random number, one of the 10 groups was selected as the source of the corresponding value for that kernel element.

For example:

Index 1: Group 1: 302.21829

Index 2: Group 3: 298.04075

Index 3: Group 2: 327.66732

Index 4: Group 5: 335.23416

...

After obtaining all 1024 R values using this randomized sampling, they were linearly converted back into weight space using the inverse mapping function.

This entire process was repeated five times, generating five distinct noisy kernels.

These kernels were used to perform face detection simulations and generate corresponding ROC curves, as shown in Figure 6h. The AUC value of each ROC curve is as follows:

| Kernel | Original | Measured 1 | Measured 2 | Measured 3 | Measured 4 | Measured 5 | Measured 6 |
| --- | --- | --- | --- | --- | --- | --- | --- |
| AUC | 0.9199 | 0.9192 | 0.9170 | 0.9201 | 0.9156 | 0.9193 | 0.9185 |

This approach reflects a realistic scenario where each element in the device array experiences independent programming noise, thus more faithfully modeling hardware variability compared to uniform noise assumptions.

**References**

1 Sun, Y. *et al.* Mesoscopic sliding ferroelectricity enabled photovoltaic random access memory for material-level artificial vision system. *Nature Communications* **13**, 5391 (2022).

2 Li, T. *et al.* Reconfigurable, non-volatile neuromorphic photovoltaics. *Nature Nanotechnology* **18**, 1303-1310 (2023).

3 Wu, G. *et al.* Ferroelectric-defined reconfigurable homojunctions for in-memory sensing and computing. *Nature Materials* **22**, 1499-1506 (2023).

4 Gong, Y. *et al.* Reconfigurable and nonvolatile ferroelectric bulk photovoltaics based on 3R-WS_2_ for machine vision. *Nature Communications* **16**, 230 (2025).

5 Zhou, Y. *et al.* Computational event-driven vision sensors for in-sensor spiking neural networks. *Nature Electronics* **6**, 870-878 (2023).

6 Zhang, G.-X. *et al.* Broadband sensory networks with locally stored responsivities for neuromorphic machine vision. *Science Advances* **9**, eadi5104 (2023)

7 Mennel, L. *et al.* Ultrafast machine vision with 2D material neural network image sensors. *Nature* **579**, 62-66 (2020).

8 Lin, H. *et al.* In situ training of an in-sensor artificial neural network based on ferroelectric photosensors. *Nature Communications* **16**, 421 (2025).

9 Cui, B. *et al.* Ferroelectric photosensor network: an advanced hardware solution to real-time machine vision. *Nature Communications* **13**, 1707 (2022).

10 Lee, S., Peng, R., Wu, C. & Li, M. Programmable black phosphorus image sensor for broadband optoelectronic edge computing. *Nature Communications* **13**, 1485 (2022).

11 Pi, L. *et al.* Broadband convolutional processing using band-alignment-tunable heterostructures. *Nature Electronics* **5**, 248-254 (2022).

12 Fu, X. *et al.* Graphene/MoS_2−x_O_x_/graphene photomemristor with tunable non-volatile responsivities for neuromorphic vision processing. *Light: Science & Applications* **12**, 39 (2023).

13 Xu, L. *et al.* Ultrasensitive dim-light neuromorphic vision sensing via momentum-conserved reconfigurable van der Waals heterostructure. *Nature Communications* **15**, 9011 (2024).

14 Dang, Z. *et al.* Object motion detection enabled by reconfigurable neuromorphic vision sensor under ferroelectric modulation. *ACS Nano* **18**, 27727-27737 (2024).

15 Wang, C.-Y. *et al.* Gate-tunable van der Waals heterostructure for reconfigurable neural network vision sensor. *Science Advances* **6**, eaba6173 (2020).

16 Huang, P.-Y. *et al.* Neuro-inspired optical sensor array for high-accuracy static image recognition and dynamic trace extraction. *Nature Communications* **14**, 6736 (2023).

17 He, Z. *et al.* Perovskite retinomorphic image sensor for embodied intelligent vision. *Science Advances* **11**, eads2834 (2025).
